# Quantitative MNase-seq accurately maps nucleosome occupancy levels

**DOI:** 10.1186/s13059-019-1815-z

**Published:** 2019-09-13

**Authors:** Răzvan V. Chereji, Terri D. Bryson, Steven Henikoff

**Affiliations:** 1 Division of Developmental Biology, Eunice Kennedy Shriver National Institute for Child Health and Human Development, National Institutes of Health, Bethesda, MD 20892 USA; 2 Howard Hughes Medical Institute and Basic Sciences Division, Fred Hutchinson Cancer Research Center, Seattle, WA 98109 USA

**Keywords:** Chromatin, Linker DNA, Nuclease digestion kinetics

## Abstract

Micrococcal nuclease (MNase) is widely used to map nucleosomes. However, its aggressive endo-/exo-nuclease activities make MNase-seq unreliable for determining nucleosome occupancies, because cleavages within linker regions produce oligo- and mono-nucleosomes, whereas cleavages within nucleosomes destroy them. Here, we introduce a theoretical framework for predicting nucleosome occupancies and an experimental protocol with appropriate spike-in normalization that confirms our theory and provides accurate occupancy levels over an MNase digestion time course. As with human cells, we observe no overall differences in nucleosome occupancies between *Drosophila* euchromatin and heterochromatin, which implies that heterochromatic compaction does not reduce MNase accessibility of linker DNA.

**Electronic supplementary material:**

The online version of this article (10.1186/s13059-019-1815-z) contains supplementary material, which is available to authorized users.

## Introduction

Nucleosomes are the basic units of DNA compaction and the fundamental constituents of chromatin [1]. They contain 147 bp of DNA, wrapped around a histone octamer comprising two copies of the four core histones: H2A, H2B, H3, and H4 [2]. DNA binding of multiple proteins is generally hindered by nucleosomes, and nucleosome positions and levels of occupancy are a common determinant of gene regulation [3–6]. Therefore, nucleosome occupancy and accessibility are important aspects of chromatin organization, with direct implications for gene regulation and all other DNA-related processes in the cell nucleus. Most yeast genes and active genes in higher eukaryotes share a stereotypical nucleosome organization of their promoters: a nucleosome-depleted region (NDR) near the transcription start site (TSS), flanked by regular arrays of nucleosomes [6–9]. This stereotypical organization has been explained by the presence of barrier complexes that bind to promoters and create an energy barrier preventing nucleosome formation [10, 11], but other determinants of nucleosome positions are also known, e.g., chromatin remodelers and DNA sequence [12].

Different cells can have different nucleosome organizational features [9, 13], and nucleosomes can occupy a given position in some cells but not others. The presence or absence of a nucleosome over a regulatory region dictates whether transcription factors may bind to the specific region or not. To obtain a quantitative understanding of gene regulation, it is extremely important to know not only the precise positions of nucleosomes, but also the percentage of cells that contain a nucleosome at a given position (nucleosome occupancy).

The most frequently used method of mapping nucleosome positions and occupancy involves digestion of chromatin with micrococcal nuclease (MNase), an endo- and exo-nuclease that preferentially digests the naked DNA between nucleosomes, releases the nucleosomes from chromatin, and enriches the nucleosome-protected DNA fragments [14]. To determine nucleosome positions and occupancy [12], the resulting undigested DNA is subjected to high throughput sequencing (MNase-seq) and mapped to the reference genome [15, 16].

Unfortunately, MNase has a sequence bias, and it cleaves DNA about 30 times faster upstream of an A or T than it does 5′ of a G or C [17, 18]. Therefore, the nucleosome occupancy profiles, obtained by stacking and counting all mononucleosomal reads covering each genomic locus, are seriously affected by the level of MNase digestion [19–21]. Nucleosomes occupying the regions of the genome which are more accessible to MNase are released faster from chromatin, and they are enriched in the mildly digested samples, while nucleosomes located in less accessible regions are released more slowly from chromatin, and they are underrepresented in the mildly digested samples but enriched in extensively digested samples [19–21].

Therefore, a quantitative interpretation of the MNase-seq occupancy profiles is still problematic. While nucleosome occupancy should be understood as the precise fraction of cells that contain a nucleosome at a given genomic locus [12], the number of nucleosomal sequences that are obtained in MNase-seq experiments depends on the level of digestion that was used in each case. This “apparent” nucleosome occupancy is a function of the level of digestion, and thus cannot be used as a proxy for the real nucleosome occupancy, which is an intrinsic property of the chromatin organization in a population of cells and must not depend on the method of investigation that is used to observe it.

Important efforts have been made recently to distinguish between nucleosome occupancy and chromatin accessibility [21, 22]. Using a linear fit of the fragment frequency (i.e., nucleosome count) that is obtained at each location, *N*, as a function of the logarithm of the MNase enzyme concentration used in the digestion, *E*:

1 $$ N=a\log (E)+b, $$

where *a* represents the slope, and *b* represents the *y*-intercept of the fit. Mieczkowski et al. have defined an MNase accessibility score, called *MACC* [21], which is effectively the negative of the fitted slope, $MACC=-a=\frac{dN}{d\left(\log E\right)}$. These authors have identified two scenarios of chromatin digestion in response to MNase titrations. If the number of nucleosomal fragments obtained from a given locus increases with the MNase concentration (*a* > 0), then this locus was characterized by a negative *MACC* score and was called “inaccessible chromatin.” If the number of nucleosomal fragments decreases with the MNase concentration (*a* < 0), then this locus was characterized by a positive *MACC* score and was labeled as “accessible chromatin.” As an alternative to the above classification given by Mieczkowski et al., another laboratory has separated the nucleosomes into “labile” (MNase-sensitive) and “stable,” by whether the nucleosomes were lost or not upon moderate or high MNase digestion [23].

There are other methods of mapping nucleosomes that are not affected by the MNase biases, e.g., sonication ChIP-seq and chemical cleavage mapping. Unfortunately, these methods present other inconveniences compared to MNase-seq. The resolution of sonication ChIP-seq experiments can be reduced to about 200 bp by using an extensive chromatin fragmentation procedure [24], but higher resolution maps are hard to obtain. Moreover, sonication has been shown to have its own biases, and heterochromatic regions are typically more resistant to fragmentation [25]. Whereas the chemical cleavage method [10, 26] offers extremely high resolution for mapping nucleosomes in small genomes, it requires mutating or downregulating and replacing the histone genes [8], which in multi-cellular eukaryotes with large genomes is complicated because they contain many copies of histone genes that are both transcriptionally and post-transcriptionally regulated [27].

Because of the various drawbacks of other methods, we focused on understanding the results produced by MNase-seq experiments in a quantitative way. By considering the kinetics of nucleosome release from chromatin by MNase, we have developed a rigorous method for analyzing MNase digestion time course experiments to calculate the nucleosome occupancy and chromatin accessibility. Our predictions are not affected by the MNase biases for individual DNA sequences nor by the variability observed at different levels of chromatin digestion in the traditional MNase-seq experiments.

Our theoretical framework shows that using Eq. (1) to characterize the chromatin accessibility may be too simplistic, as mononucleosome counts are not predicted to follow a single slope obtained from Eq. (1) throughout the entire digestion course. In the initial stages of chromatin digestion in a population of cells, the number of nucleosomes that are released from every genomic locus will increase while the digestion proceeds, i.e., increased chromatin fragmentation results in more mononucleosomes that are released from multiple cells. However, in the later stages of digestion, after most of chromatin has been reduced to mononucleosomal fragments, continuing the digestion even further reduces the number of intact nucleosomes that are recovered from any locus, as MNase starts to over-digest and destroy the intact nucleosome core particles. Therefore, the amount of intact nucleosome fragments that are obtained in MNase-seq experiments from any genomic locus cannot be accurately modeled by a monotonic function of the level of digestion. Instead, the number of nucleosomes should be modeled by a function that initially increases from 0 to a maximum level, after which it slowly decreases until it vanishes again, at the stage of digestion when all intact nucleosomes have been destroyed by MNase.

Here, we analyze the process of chromatin digestion by MNase in a quantitative way, and we show that different properties of chromatin, such as nucleosome occupancy and accessibility of different genomic loci, can be disentangled using classical kinetics modeling of the biochemical reactions involving MNase and chromatin. To test the prediction of our theoretical model for chromatin digestion, we have developed a quantitative MNase-seq (q-MNase-seq) method, which allows for meaningful comparisons between the number of mononucleosomes that are obtained genome-wide at different levels of digestion, unlike the traditional MNase-seq method.

## Results

### Kinetics of chromatin digestion and nucleosome release by MNase

It has been shown that a brief digestion by MNase produces globular supranucleosomal structures that may contain a large number (8–48) of nucleosomes [28]. The existence of these resistant domains suggests that the accessibility of MNase to different regions of the genome is variable, and different nucleosomes may be released from chromatin at different rates. For example, nucleosomes from unfolded arrays could be released from chromatin faster than nucleosomes forming the compact supranucleosomal structures. Moreover, the very characteristic “nucleosome ladder” pattern observed in gel electrophoresis of DNA resolved from MNase-digested chromatin suggests that different regions of the genome may be digested by MNase at different rates: while some parts of chromatin are only broken into large fragments, other parts are reduced to mononucleosome-size fragments.

**Fig. 1:**
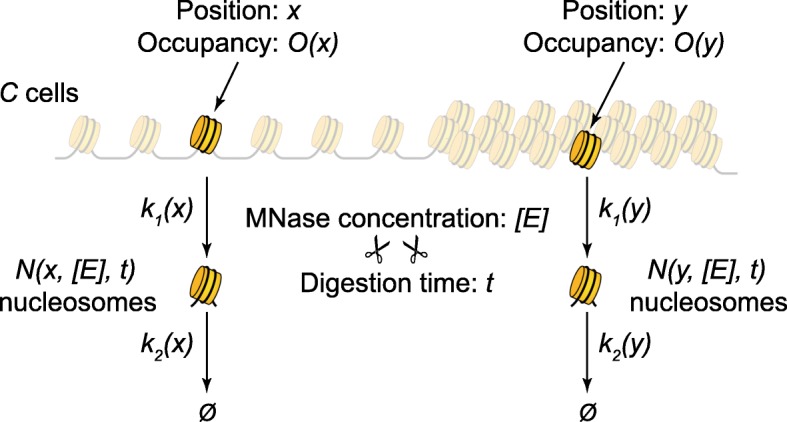
Kinetics of nucleosome release from chromatin. Each position *x* along the genome is characterized by a nucleosome occupancy (i.e., the fraction of cells in which position *x* is occupied by a nucleosome), a rate of nucleosome release (rate constant *k*_1_(*x*)), and a rate of nucleosome decay (over-digestion, rate constant *k*_2_(*x*)). Digesting a population of *C* cells with a concentration [*E*] of MNase, for a time *t*, will result in a number *N*(*x*, [*E*], *t*) of free mononucleosomes from each position *x* (Additional file 1). Nucleosomes from the more accessible regions of chromatin (*x*) may be released faster than nucleosomes from the regions of less accessible chromatin (*y*)

To account for the fact that different regions of chromatin may be digested by MNase at different rates, we have developed a rigorous kinetic theory of nucleosome release from chromatin (Additional file 1). We model the chromatin digestion as shown in Fig. 1. Each genomic locus is characterized by a nucleosome occupancy (i.e., the fraction of cells in which this locus is occupied by a nucleosome) and a nucleosome accessibility. The more accessible nucleosomes are the ones that are released first by MNase from chromatin.

Suppose we start with a population of *C* cells, and we focus on a particular genomic locus *x*. Let us denote the fraction of cells that contain a nucleosome (i.e., nucleosome occupancy) at this locus by *O*(*x*). Therefore, among the *C* cells, only a number of *CO*(*x*) cells contain a nucleosome occupying position *x*, and the rest of *C*[1 − *O*(*x*)] cells contain a linker at position *x* in their genome. At the beginning of the MNase digestion reaction, all *CO* nucleosomes are part of intact chromatin fibers, and we denote the number of “bound” nucleosomes at time *t* = 0 by *B*(0). As digestion proceeds, after time *t*, we will have a number of nucleosomes, *B*(*t*), that are still bound to chromatin and a number of nucleosomes, *N*(*t*), that had already been released from chromatin as mononucleosomes. During digestion, nucleosomes are released from chromatin, increasing *N*(*t*) and decreasing *B*(*t*). At the same time, MNase also attacks free mononucleosomes from the sample, slowly decaying the number of these due to over-digestion and internal cleavages followed by nucleosome disassembly. These two processes can be represented by a simple reaction chain, *B* → *N* → *Ø*. The corresponding concentrations of nucleosomes from the sample satisfy the following differential equations:

$$ \frac{d\left[B\right]}{dt}=-{k}_1\left[B\right]\left[E\right] $$

$$ \frac{d\left[N\right]}{dt}={k}_1\left[B\right]\left[E\right]-{k}_2\left[N\right]\left[E\right] $$

where [*E*] is the concentration of free MNase, which remains approximately constant in the experiment, and *k*_1_ and *k*_2_ represent the rate constants for the nucleosome release and decay reactions, respectively (Additional file 1). The solutions to these equations, satisfying the initial conditions *B*(0) = *CO* and *N*(0) = 0 are (Additional file 1):

2 $$ {\displaystyle \begin{array}{c}\left[B\right](t)=\left[C\right]{Oe}^{-{k}_1\left[E\right]t}\\ {}\left[N\right](t)=\left[C\right]O\frac{k_1}{k_1-{k}_2}\left({e}^{-{k}_2\left[E\right]t}-{e}^{-{k}_1\left[E\right]t}\right)\end{array}} $$

Therefore, after time *t*, a fraction ${f}_B(t)=\frac{\left[B\right](t)}{\left[B\right](0)}$ of the nucleosomes initially found at locus *x* in the population of cells are still bound to the chromatin fibers and a fraction ${f}_N(t)=\frac{\left[N\right](t)}{\left[B\right](0)}$ of the nucleosomes were released from chromatin and are present as intact mononucleosomes in the sample. Additional file 1: Figure S1 shows the dependence of these nucleosome fractions on the level of digestion, denoted by *d* = [*E*]*t*. As seen in Additional file 1: Figure S1, the predicted fraction of mononucleosomes that are present in the sample (red line) does not vary monotonically with the degree of digestion [21] but has a more complex shape. The nucleosome counts will initially increase with the level of digestion, until they reach a maximum, for a digestion level at which the rate of nucleosome release is equal to the rate of nucleosome decay, *k*_1_[*B*][*E*] = *k*_2_[*N*][*E*], which is equivalent to ${k}_1{e}^{-{k}_1\left[E\right]t}={k}_2{e}^{-{k}_2\left[E\right]t}$ (Additional file 1). If digestion continues further, the rate of nucleosome decay, (*k*_2_[*N*][*E*]), starts to be higher than the rate of release of new nucleosomes from chromatin (*k*_1_[*B*][*E*]), and the overall level of intact mononucleosomes starts to decrease until it completely vanishes, when MNase has destroyed all nucleosomes by over-digestion.

Different genomic loci *x* are characterized by different nucleosome occupancies and accessibilities, and the nucleosome counts that are obtained from each locus will depend on four parameters: the level of digestion *d*, nucleosome occupancy *O*(*x*), and the two rate constants *k*_1_(*x*) and *k*_2_(*x*), corresponding to the nucleosome release and decay processes, respectively. The predicted dependence of the nucleosome counts obtained from any genomic locus on the corresponding parameters *O*, *k*_1_, *k*_2_, and on the level of digestion *d*, is shown in Additional file 1: Figure S2 and S3. Additional file 1: Figure S2a shows that nucleosome occupancy *O* affects the overall height and area of the nucleosome count profile as a function of the digestion level, acting as a simple rescaling factor when the other parameters (*k*_1_, *k*_2_) are kept constant. Additional file 1: Figure S2b,c shows that the rate constant *k*_1_ mostly affects the initial slope of the apparent occupancy, *N*/*C*, while the decay rate constant *k*_2_ mostly affects the right tail of the apparent occupancy distribution. The fact that the three parameters *O*, *k*_1_, and *k*_2_ influence different aspects of the apparent occupancy (Additional file 1: Figure S2) guarantees the uniqueness of the parameters obtained by fitting this distribution using Eq. (2). Additional file 1: Figure S3 reinforces this conclusion by showing that the rate constant *k*_2_ can be precisely obtained from the asymptotic behavior of the logarithm of the apparent occupancy for extensive digestions, and the estimated values of *k*_2_ are not affected by the other two parameters (*O*, *k*_1_).

Although the solution given by Eq. (2) seems complicated, one can easily use a non-linear fit algorithm in order to obtain the parameters *O*, *k*_1_, and *k*_2_, if the nucleosome amounts generated at multiple digestion levels are measured. For example, this non-linear fit can be obtained using the lsqcurvefit function in MATLAB, nls function in R, or scipy.optimize.leastsq function in Python.

Unfortunately, the traditional MNase-seq method is not appropriate for a rigorous comparison and fit of the nucleosomal counts that are obtained in different experiments. In general, when MNase-seq data are compared, these data are normalized such that the total number of reads in all experiments is set to a common value. This normalization is not valid for our purpose, as the total number of nucleosomes released from chromatin depends on the level of digestion. For this reason, we adopted a modified MNase-seq procedure, q-MNase-seq (Additional file 1: Figure S4), which allows us to do meaningful comparisons between the nucleosome counts obtained at different stages of the digestion and to fit the data in order to obtain the three parameters (*O*, *k*_1_, *k*_2_) for each nucleosome, as described next.

### Quantitative measurement of the number of nucleosomes released from chromatin at different levels of digestion

We start with similar numbers of *Drosophila* S2 cells obtained by splitting the total culture into equal volumes. These aliquots are subjected to different levels of digestion by MNase. The resulting undigested DNA is then purified. Since each sample will release a different number of nucleosomes, depending on the corresponding degree of digestion, to keep track of the relative amounts of mononucleosomes from all samples, we add equal amounts of spike-in DNA (*Saccharomyces cerevisiae* mononucleosomal DNA obtained by a traditional MNase-seq method). Then, each sample consisting of a mix of fly and yeast DNA is used to prepare the sequencing library. As each sample already contains the spike-ins, it is acceptable to use different numbers of cycles of PCR amplification, if that is necessary in order to obtain similar amounts of total DNA for sequencing. After sequencing, the DNA from each sample is aligned both to the *Drosophila* genome (version *dm6*) and to the *S. cerevisiae* genome (version *sacCer3*) to obtain the numbers of fly mononucleosomes and spike-in reads. Then, we normalize the counts of fly mononucleosomes by the amount of spike-in DNA from each sample such that all samples have the same number of spike-in fragments. Using the normalized data, we can analyze any genomic region and plot the nucleosome counts as a function of the digestion level (Additional file 1: Figure S4). These data can be fitted using Eq. (2) to obtain the nucleosome occupancy, *O*, and the two rate constants, *k*_1_ and *k*_2_, for each genomic locus (Additional file 1: Figure S5).

**Fig. 2:**
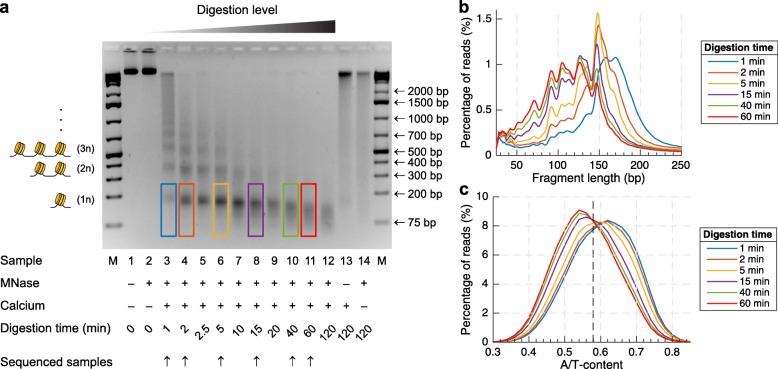
The level of chromatin digestion determines the set of nucleosomes that are obtained by MNase-seq. **a** Electrophoresis of MNase-digested chromatin shows the characteristic “nucleosome ladder” pattern, which contains bands corresponding to mononucleosomes (1n), di-nucleosomes (2n), tri-nucleosomes (3n), and longer oligonucleosome fragments. **b** The length distributions of the mononucleosomal bands are marked by colored rectangles on the gel. Increasing the level of digestion reduces the undigested footprints of the nucleosomes. **c** These nucleosomal DNA fragments originate from different sets of nucleosomes, as illustrated by the difference in their A/T content distribution. The set of nucleosomes obtained in the early stages of digestion is originating from more A/T-rich regions compared to the set of nucleosomes obtained in the later stages of digestion. The average A/T content of the *Drosophila* genome is ~ 58% and is marked by the vertical dashed line

We performed a q-MNase-seq experiment, by digesting *Drosophila* S2 cells with equal amounts of MNase for various time periods: 0 min (control), 1 min, 2 min, 2.5 min, 5 min, 10 min, 15 min, 20 min, 40 min, 60 min, and 120 min. The extent of digestion was verified by gel electrophoresis in an agarose gel (Fig. 2a). In theory, three time points are required to fit the three parameters, *O*, *k*_1_, and *k*_2_, but because of stochastic noise, five to six time points are preferred to obtain sufficient accuracy. We selected six time points (1 min, 2 min, 5 min, 15 min, 40 min, 60 min) for which we performed end-polishing, adapter ligation, PCR amplification, and paired-end sequencing of the undigested DNA fragments without size selection. As previously observed [20], the mononucleosomal bands from the mild digestions contain longer DNA fragments (Fig. 2b) originating from the more A/T-rich regions of the genome (Fig. 2c), while the mononucleosomal bands from the more extensive digestions contain shorter DNA fragments (Fig. 2b), and correspond to nucleosomes that occupy the more G/C-rich regions of the genome (Fig. 2c).

**Fig. 3:**
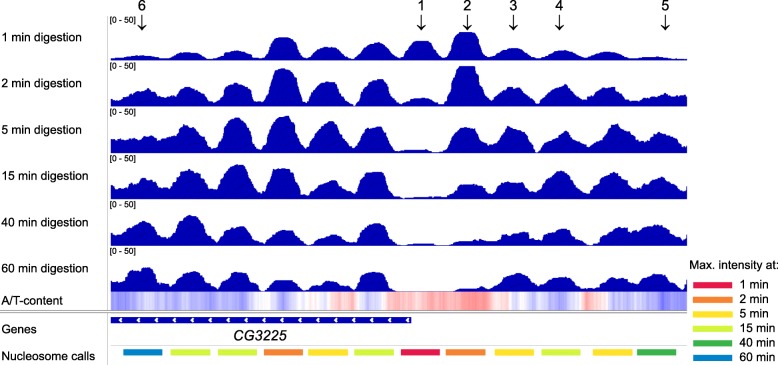
Nucleosome counts depend on the level of digestion and are not proportional to nucleosome occupancy. IGV browser snapshot (chromosome 2L: 4,817,130–4,819,270) showing the normalized mononucleosomal read counts that are obtained after digesting the nuclei with MNase for various intervals of time (indicated on the left side of each track). The seventh track, shown as a heat map, represents the variation of A/T content along the genome. White color represents the genome-wide average A/T content (~ 0.58), red color represents A/T-rich regions, and blue color represents G/C-rich regions. The identified peaks and their classification are shown in the last track. Note that peaks that disappear very rapidly (e.g., 1) may correspond to non-histone complexes, such as nucleosome remodelers [29]

The nucleosome counts that were obtained in the six experiments were then normalized by the spike-in DNA amounts from each sample. An IGV browser snapshot of a representative region of the genome illustrates some of the differences among the properly normalized nucleosome counts observed after different levels of digestion (Fig. 3). The nucleosome counts vary with the level of digestion for each nucleosome. Some nucleosomes are quickly released from chromatin (Fig. 3, peaks 1 and 2), and they are enriched in the samples that were just mildly digested for 1 or 2 min. Peak 1 in Fig. 3 likely represents a non-histone barrier complex [20] that offers only a limited protection against MNase or a RSC-nucleosome complex [29] that is observed in a small number of cells during the remodeling process. Such nucleosomes are also the first ones that are over-digested by MNase and are underrepresented or completely disappear from the distributions obtained in the latest stages of the digestion (40 min, 60 min). These kinds of nucleosomes are usually denoted as MNase-sensitive nucleosomes and were shown to contain A/T-rich sequences [19, 20]. In yeast and animals, active regulatory elements are often adjacent to asymmetrically unwrapped nucleosomes [30–32]. In yeast, these nucleosomes are generally more accessible to MNase due to the RSC complex being bound to them [29], which destabilizes the nucleosome core particle and exposes more DNA to MNase.

Using the combined nucleosome counts from the six experiments, we next identified the typical positions of nucleosomes along the *Drosophila* genome, using a custom algorithm for computing the median position for all clusters of aligned nucleosomes (described in Additional file 1). We identified about 700,000 typical nucleosome positions, covering about 73% (100 Mb) of the whole genome size. The expected fraction of the *Drosophila* genome that is covered by nucleosomes is roughly 73%, given that each nucleosome covers 147 bp out of every 200 bp (the typical nucleosome repeat length in flies). Therefore, we are confident that our nucleosome calling algorithm has detected most of the nucleosomes, including the poorly positioned ones.

In order to offer enough protection against MNase to result in a peak in MNase-seq occupancy profiles, a protein must be stably bound to DNA. The transient in vivo binding of fast transcription factors will not protect DNA during 5–60 min time course of MNase digestion. Such transient binding events may only give a significant peak in MNase-seq occupancy profiles obtained by digesting the chromatin for a very short period of time (e.g., 1 min digestion in our experiments). As we describe below, the sites that were bound by “MNase-sensitive” complexes (i.e., MNase-seq peaks that were observed in the mild digestions and disappeared in the latter stages of digestion) accounted for fewer than 1% of the total number of identified peaks.

The bottom track in Fig. 3 shows the identified nucleosomes in the corresponding region near the gene *CG3225*. After we identified the well-positioned nucleosomes and normalized the nucleosome counts using the spike-in levels from each sample, we were able to study the evolution of the number of released nucleosomes as a function of the digestion level. Even in a small region of the genome (Fig. 3), neighboring nucleosomes are released with different kinetics, e.g., peak 1 has its maximum intensity after 1 min of digestion, peak 2 after 2 min of digestion, peak 3 after 5 min of digestion, peak 4 after 15 min of digestion, peak 5 after 40 min of digestion, and peak 6 after 60 min of digestion.

In another region of the *Drosophila* genome, the gene start and end sites are A/T-rich (Additional file 1: Figure S6), as is usually the case for many genes in diverse organisms. These loci are occupied by MNase-sensitive nucleosomes, which are underrepresented in the latest stages of the digestion. Again, A/T-rich regions that contain nucleosomes are released faster (colored in red, orange, and yellow), while G/C-rich regions that contain nucleosomes are released later in the digestion process (colored in green and blue).

Traditionally, MNase-seq experiments are performed using extensive levels of digestion (when > 80% of the chromatin is reduced to mononucleosomes). It was previously assumed that nucleosome gaps at promoters and transcription termination regions are largely nucleosome-free [6]. We believe that this interpretation does not hold for transcription termination sites (TTSs), which are some of the most A/T-rich regions in the genome, and therefore, nucleosomes are artificially underrepresented from these loci in the extensively digested samples [10, 20]. Moreover, promoters are also A/T-rich regions, and nucleosomes from these regions may also be underrepresented in the typical MNase-seq experiments, usually corresponding to a fairly extensive level of digestion. For this reason, if an extended A/T-rich intergenic region appears to be depleted of nucleosomes in the typical MNase-seq experiments, it must be remembered that this could be just an artifact introduced by an extensive level of MNase digestion.

### Nucleosomes occupying different DNA sequences are released from chromatin at different rates

**Fig. 4:**
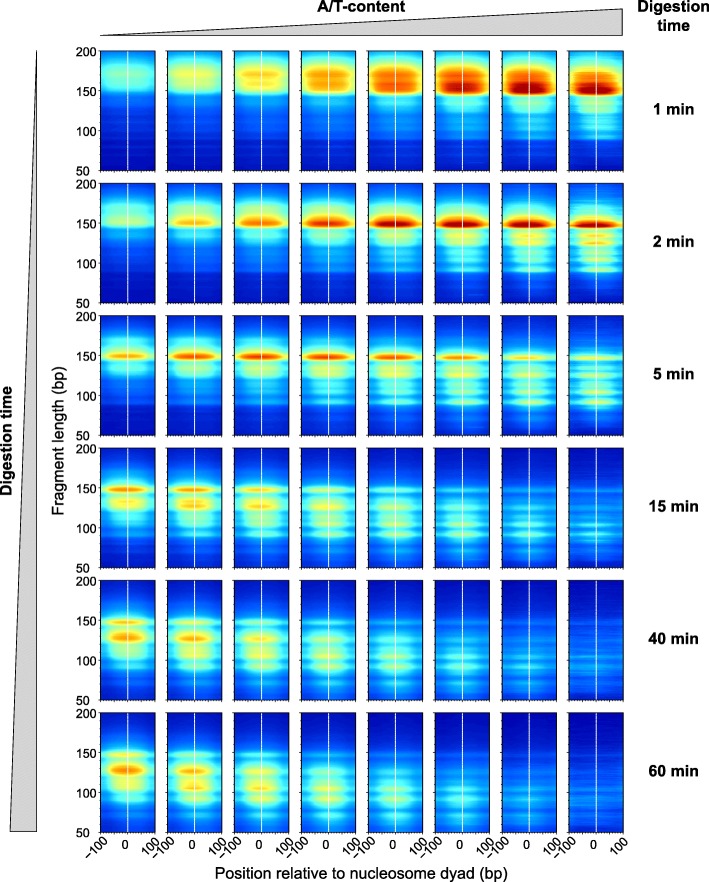
2D occupancy plots indicate the fraction of nucleosomes obtained during the digestion from regions with different A/T content. About 700,000 nucleosomes were grouped according to their A/T content into 8 bins (0–0.5, 0.5–0.55, 0.55–0.6, 0.6–0.65, 0.65–0.7, 0.7–0.75, 0.75–0.8, 0.8–1), and for each bin, we show the distribution of fragment sizes obtained at different levels of digestion. Digestion times, from top to bottom: 1 min, 2 min, 5 min, 15 min, 40 min, 60 min. 2D occupancy plots were generated using the *plot2do* package available on GitHub at https://github.com/rchereji/plot2DO

From Fig. 3 and Additional file 1: Figure S6, one can hypothesize that nucleosomes found in A/T-rich regions are released faster from chromatin compared to nucleosomes found in G/C-rich regions. Those that are released faster could also be destroyed faster and lost from the mononucleosomal band of the extensively digested samples. We next tested this hypothesis using all the well-positioned nucleosomes that we identified. Figure 4 shows the density of sequencing reads that we obtained for each time point of the digestion, as a function of the A/T content of these nucleosomes. We see that in the initial stages of the digestion (Fig. 4, top row) the A/T-rich nucleosomes are relatively over-represented in the sample, and the mononucleosomal fragments are slightly longer than 147 bp, indicating that linker DNA adjacent to the nucleosome core particles is not yet fully trimmed. When digestion proceeds further, MNase starts to invade the nucleosome core, resulting in shorter DNA fragments (< 147 bp). The A/T-rich nucleosomes (the right-most bin) that were released first from chromatin are almost completely destroyed by MNase after 40 min of digestion (bottom two rows, 40 min and 60 min, respectively). In the latest stages of digestion (bottom row), the A/T-rich nucleosomes are underrepresented from the sample and contrary to the mild digestion (top row); now, it is the G/C-rich nucleosomes that are relatively over-represented in the sample.

Because A/T-rich nucleosomes are destroyed first (Fig. 4) and are underrepresented in the extensively digested samples (Fig. 4, bottom row), it was previously claimed that nucleosomes have an intrinsic preference for the G/C-rich sequences [33], and models have been proposed to explain nucleosome organization based on DNA sequence alone [34, 35]. We believe that the G/C enrichment in the nucleosomal DNA that was previously observed may be an artifact introduced by MNase, which preferentially destroyed most of the A/T-rich nucleosomes by the time the digestion reaction was stopped. Moreover, any computational model that was trained with the nucleosome occupancy profiles obtained from extensively digested chromatin, could be artificially biased to favor the G/C-rich locations on the genome and would underestimate the A/T-rich nucleosomes.

### The experimental data confirms the prediction of our theoretical framework

**Fig. 5:**
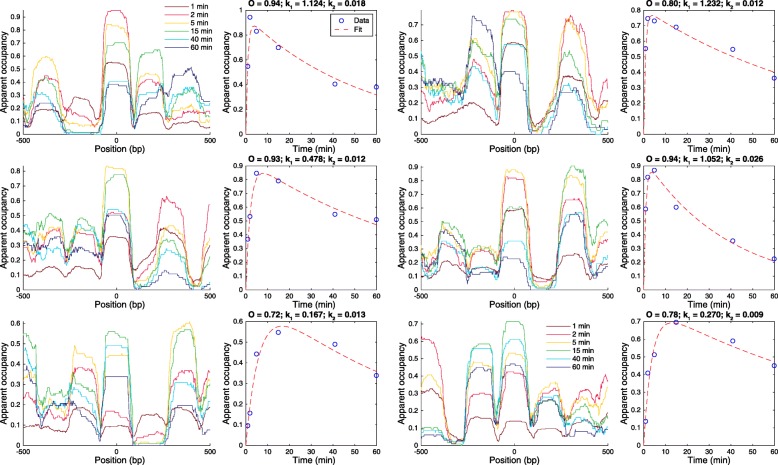
Normalized coverage (apparent occupancy) profiles confirm the prediction of the theoretical model. Apparent occupancy profiles for six regions of the genome (left panels) and the corresponding quantification of the average occupancy for the nucleosomes in the center of each panel (the corresponding right panels, blue circles). The occupancy levels for all six nucleosomes (blue circles) vary during the digestion as predicted by Eq. (2). Using the predicted analytical behavior given by Eq. (2), we can fit the experimental data very well, and we obtain the corresponding three parameters that characterize the digestion kinetics of each genomic locus: nucleosome occupancy *O* and the two rate constants *k*_1_ and *k*_2_, corresponding to the nucleosome release and decay processes, respectively. The fitted parameters are displayed above the right-hand plots, and the predicted dependence of the apparent nucleosome occupancy (fraction of nucleosomes present in the sample at each time point) on the level of digestion is shown with red dashed lines. The example nucleosomes shown in the figure occupy the following positions (centers of the 1-kb windows): (top row) chr2L:19,509,018 and chr2R:22,605,540; (middle row) chr3R:24,050,605 and chr2R:11,303,325; (bottom row) chr2L:1,732,199 and chr3R:27,301,872. These six examples were chosen such that the top two cases correspond to the regions for which the corresponding nucleosome counts have maxima after 2 min of digestion, the next two cases correspond to the regions that are digested more slowly, and the corresponding maxima are observed after 5 min of digestion, and the bottom panels correspond to the regions that are digested at an even slower rate, with the corresponding maxima obtained only after 15 min of digestion

Using the spike-in normalized nucleosome counts, we can test the prediction of our theoretical model. Having identified all well-positioned nucleosomes, we analyzed the digestion time course for all ~ 700,000 regions of the genome where we identified a nucleosome. Figure 5 shows the spike-in normalized coverage (apparent occupancy) for 6 loci, centered on 2 nucleosomes that give the maximum signal after 2 min of digestion (upper panels), 2 after 5 min of digestion (center panel), and 2 after 15 min of digestion (lower panels). From the occupancy profiles (Fig. 5, left plots from each pair), we computed the average occupancy for the center nucleosome, and we plotted these as a function of the digestion time (Fig. 5, blue circles in the right plots from each pair). These values follow the predicted dependence on the level of digestion (Eq. (2)), and using Eq. (2), we can fit the data to obtain the real nucleosome occupancy *O* and the two rate constants *k*_1_ and *k*_2_ characterizing the nucleosome release and decay processes.

**Fig. 6:**
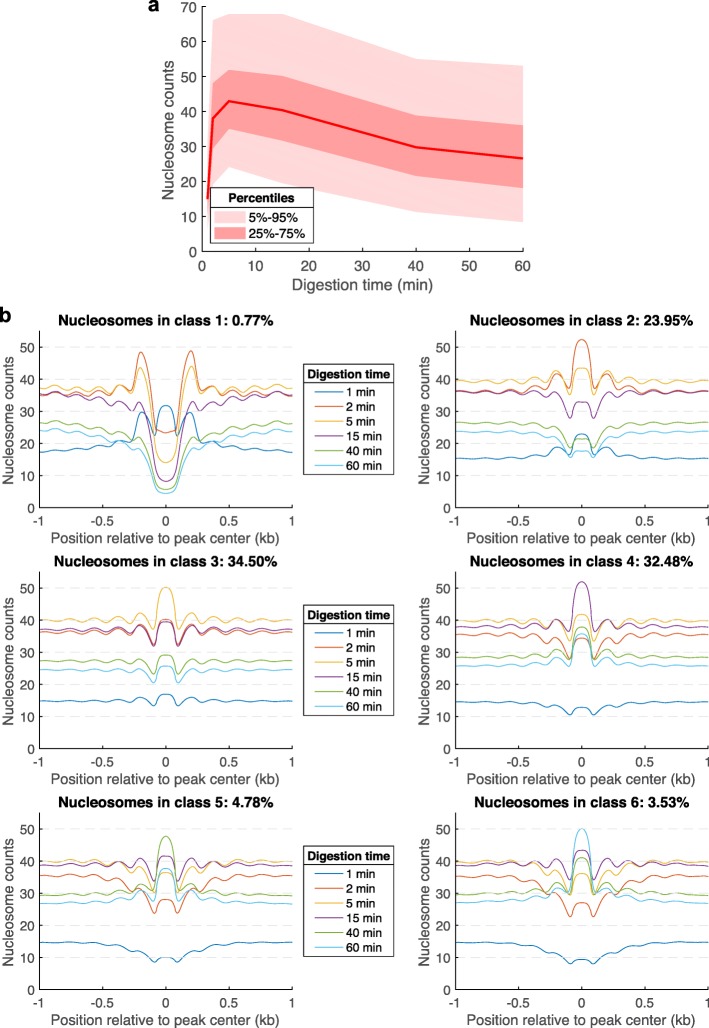
Average nucleosome counts for the six classes of nucleosomes. **a** Median nucleosome count (red line), the interquartile range (25–75%), and the 5–95% range, as a function of digestion time. **b** Average nucleosome counts for the six classes of nucleosomes that we identified according to the digestion time after which they generate the strongest signal in our experiments

Figure 6a shows the distribution of the normalized nucleosome counts of all nucleosomes as a function of the level of digestion. As predicted by our model (Additional file 1: Figures S1 and S2), the normalized nucleosome counts are initially increasing to a maximum, and then they decrease after enough nucleosomes have been released from chromatin and when the rate of nucleosome decay is higher than the rate of nucleosome release. For more than 90% of the nucleosome loci, the maximum nucleosome count is obtained after 2 min (23.95% of nucleosomes), 5 min (34.50%), or 15 min of digestion (32.48%). A small fraction of the loci (< 1%) are characterized by a maximum nucleosome count obtained after only 1 min of digestion (the first time point that we sequenced), and another small fraction of the loci (~ 3.53%) give the maximum nucleosome counts after 60 min of digestion. We grouped these loci into six classes, according to the time point at which we obtained the maximum nucleosome count at the corresponding locus. Figure 6b shows the average nucleosome counts that were obtained for all six classes of nucleosomes and all six digestion levels. Apart from all other classes, the loci from class 1 show significant protection only at the initial time point (1 min), and after that, these loci are characterized by a trough between two other nucleosomes. This class of weakly protected loci (< 1% of all called MNase-seq peaks) likely represent the MNase-sensitive complexes formed by non-histone proteins [20], which still weakly hinder MNase digestion. Figure 6 suggests that neighboring nucleosomes are not released from chromatin in an independent way, as they share a common linker. If a linker is AT-rich or particularly accessible to MNase, then both of the neighboring nucleosomes are expected to be released faster from chromatin, compared to other nucleosomes, which do not have any of the two linkers preferentially cut by MNase.

**Fig. 7:**
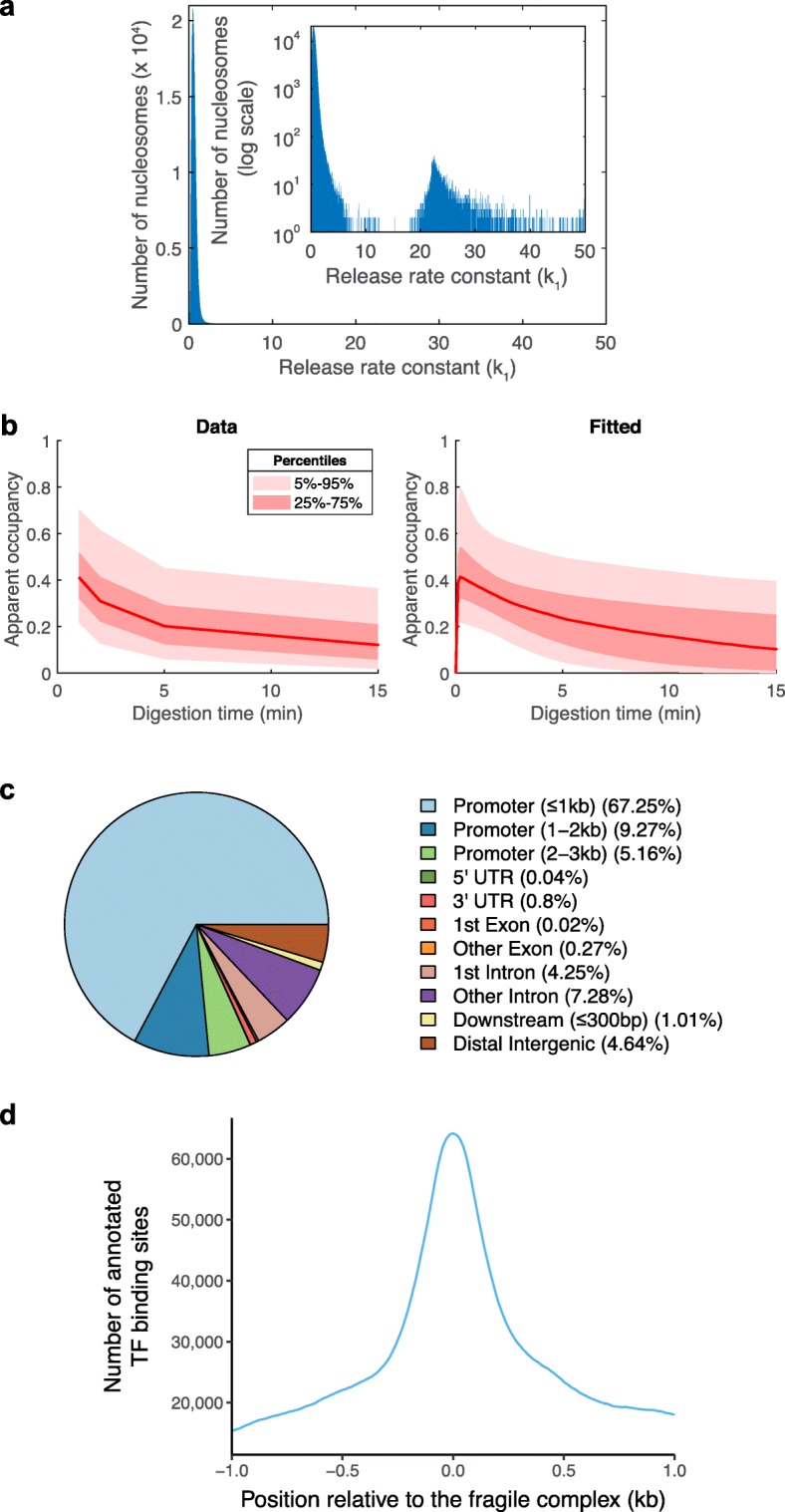
The chromatin digestion rate separates the normal nucleosomes from fragile complexes. **a** Histogram of the number of *k*_1_ values obtained by fitting the nucleosome counts using Eq. (2). A small fraction (< 1%) of the loci where we identified MNase-seq peaks are characterized by very high chromatin digestion rate constants (*k*_1_ > 10) compared to typical nucleosomes (*k*_1_ ≈ 1). **b** The rescaled nucleosome counts (apparent occupancy, $\frac{N\left(x,t\right)}{C}$) of the loci characterized by high chromatin digestion rates (*k*_1_ > 10) (left) and the corresponding fitted curves, given by Eq. (2) (right). **c** Annotations of the loci occupied by fragile complexes (*k*_1_ > 10) obtained with ChIPseeker [36]. **d** The ~ 4500 loci bound by fragile complexes contain ~ 60,000 annotated binding sites of different transcription factors (annotations of TF binding sites in S2 cells were downloaded from ChIP Atlas, https://chip-atlas.org)

Analyzing the distribution of the chromatin digestion rates obtained by fitting the nucleosome counts using Eq. (2), we observed two groups of sites (Fig. 7a). Most of the sites (> 99%) are characterized by values of *k*_1_ close to 1 and represent the typical nucleosomes, which are released from chromatin at relatively slow rates. The second group of sites (< 1%) is characterized by relatively high values of the chromatin digestion rate *k*_1_(>10) and represents loci that are bound by relatively fragile complexes, which offer only a weak protection against MNase and are quickly destroyed by MNase. Figure 7b shows the distribution of apparent occupancy for these MNase-sensitive complexes (left) and the corresponding distributions that we obtained by fitting these data using Eq. (2) (right panel). Using ChIPseeker [36], we annotated the ~ 4500 loci occupied by fragile complexes. We found that most of these loci are located in gene promoters (~ 82%), while others are located in introns (~ 11%) and intergenic regions (~ 5%) (Fig. 7c). To find out whether these sites were previously annotated in the literature or not, we have downloaded the annotations of all transcription factor (TF) binding sites that are available on ChIP Atlas (https://chip-atlas.org). As expected, we found a strong enrichment of TF binding sites at the loci occupied by fragile complexes (Fig. 7d). Therefore, these fragile complexes are likely to represent TFs bound to promoters or enhancers, or other proteins that can bind and destabilize nucleosomes, such as SWI/SNF family chromatin remodeling complexes [29].

**Fig. 8:**
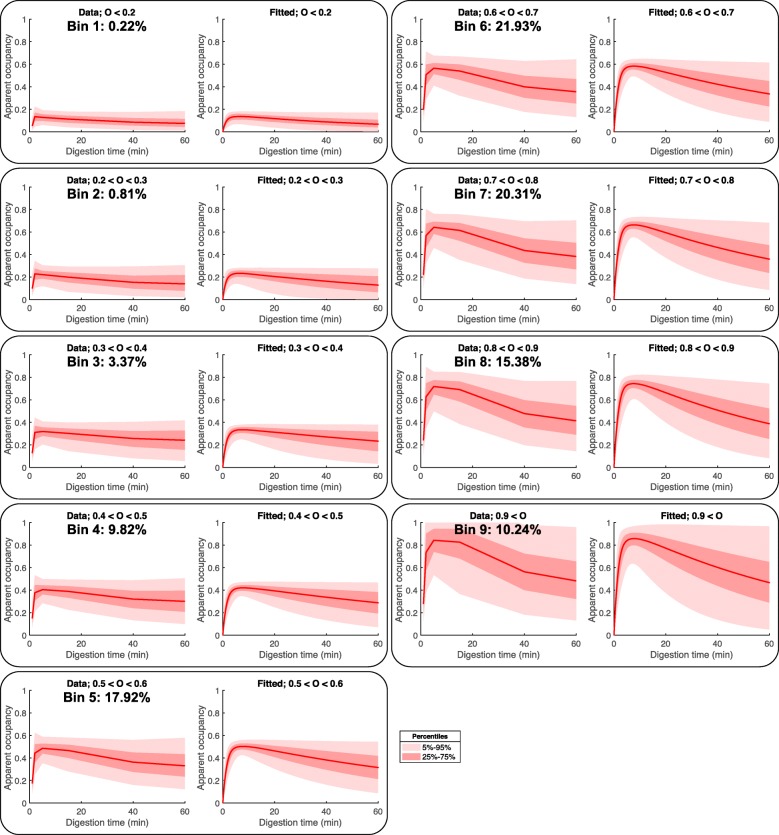
Fitting the nucleosome counts for different levels of digestion reveals a wide range of nucleosome occupancy values. About 700,000 identified nucleosomes were split into 9 bins according to the nucleosome occupancy obtained by fitting the nucleosome counts using Eq. (2). The left panels show the normalized nucleosome counts as a function of the digestion time (median, red line; percentiles, pink areas), and the right panels show the fitted apparent occupancies ($\frac{N\left(x,t\right)}{C}$)

The bulk of chromatin is occupied by nucleosomes that are released at a relatively lower rate (*k*_1_ ≈ 1) (Fig. 7a). Surprisingly, these nucleosomes are characterized by a wide range of occupancy levels (Fig. 8). Using the fitted values for the nucleosome occupancy, we split the ~ 700,000 loci along the *Drosophila* genome according to their corresponding occupancy (Fig. 8) into nine bins. Equation (2) fitted well the apparent occupancy for all groups of nucleosomes (Fig. 8), confirming that the nucleosome counts that are obtained during MNase-seq experiments can be well modeled using our theoretical framework.

### Different chromatin regions are characterized by similar nucleosome accessibilities

**Fig. 9:**
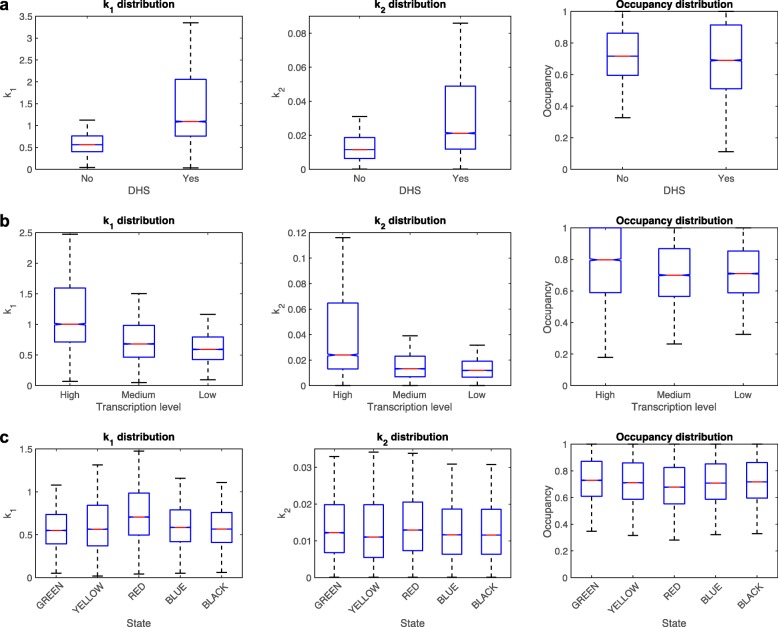
MNase accessibility to DNA is very similar for different regions of the genome. **a** Distribution of *k*_1_, *k*_2_, and *O* values for nucleosomes near DHSs and away from DHSs. **b** Distribution of *k*_1_, *k*_2_, and *O* values for the regions [TSS − 250, TSS + 250] corresponding to three gene tertiles obtained by splitting the genes by their expression levels (RNA-seq data from [19]). **c** Distribution of *k*_1_, *k*_2_, and *O* values for the five chromatin regions defined in [38]

To test whether different regions of the genome have very different chromatin accessibilities, as suggested by DNase-seq and ATAC-seq experiments, we next analyzed the distribution of nucleosome release rates that we obtained along the genome. We first analyzed the DNaseI hypersensitive regions (DHSs) (obtained from [37]) and compared the nucleosome accessibility of the nucleosomes located less than 250 bp from DHSs and those away from DHSs. As expected, nucleosome accessibility, as measured by the nucleosome release rate constant *k*_1_, is higher near DHSs (Fig. 9a, left panel), but surprisingly, the accessibility of nucleosomes from DHSs is still of the same order of magnitude as the accessibility of DNaseI insensitive regions (less than twofold difference between the median levels of *k*_1_). The middle and right panels in Fig. 9 show the distributions of the fitted *k*_2_ and nucleosome occupancy, for the nucleosomes belonging to the indicated group.

To test whether transcription affects the nucleosome accessibility, we split the genes into three tertiles according to the transcription levels obtained from RNA-seq data [19], and we compared the accessibility of the nucleosomes located near the TSSs of these genes ([TSS − 250, TSS + 250]). We observed that nucleosome accessibility correlates with transcription, and the tertile of the most transcribed genes contained the most easily accessible nucleosomes (Fig. 9b). Again, the differences were relatively small, and the overall accessibility of the promoters corresponding to the three tertiles did not vary by orders of magnitude, but only by less than twofold.

Next, we tested whether the regions of the genome that could be away from promoters contain either highly accessible or inaccessible nucleosomes. For this, we compared the accessibility of the five previously reported chromatin states, obtained by principal component analysis and clustering of genome-wide distributions of 53 chromatin proteins, mapped using the DamID method [38]. The distribution of the *k*_1_ values for the nucleosomes corresponding to the five chromatin states are shown in Fig. 9c. Only the “RED” chromatin state contains nucleosomes that are relatively more accessible, as expected since the “RED” chromatin state contains “hubs of regulatory activity” [38] and are enriched in origins of replication. Surprisingly, the “BLUE” and “GREEN” chromatin states, which contain the known heterochromatin regions, are only slightly less accessible to MNase compared to the other three chromatin states, which are predominantly euchromatic (Fig. 9c).

### Nucleosome phasing by transcription-related barrier complexes

**Fig. 10:**
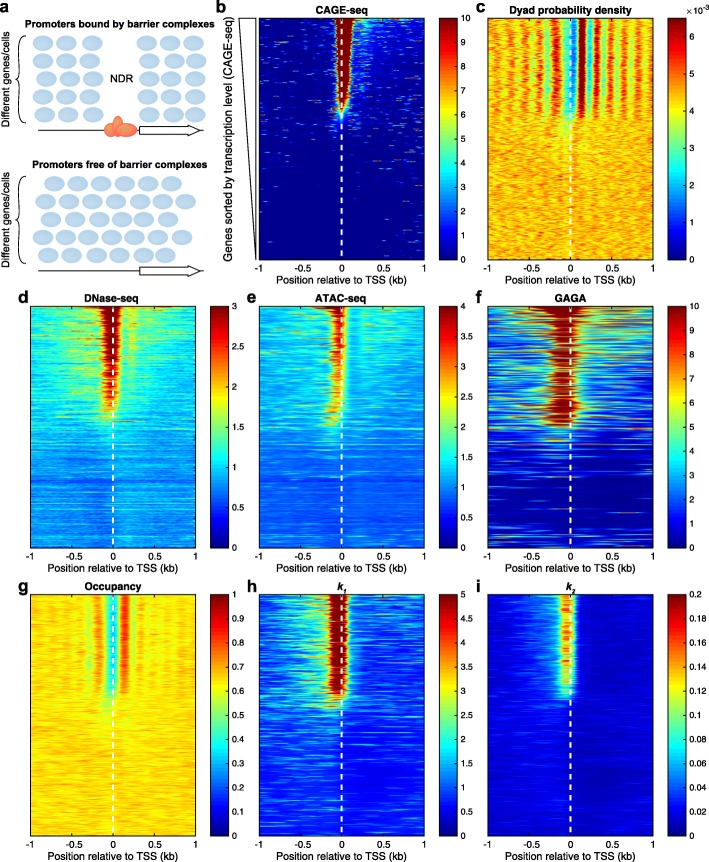
Promoter nucleosome organization: a two-state model. **a** Our model predicts two different nucleosome organization of gene promoters. Promoters bound by barrier complexes are predicted to contain an NDR flanked by phased nucleosomes, while promoters that are free of barrier complexes are predicted to have disorganized nucleosomes and lack NDRs. **b** *Drosophila* promoters sorted by the transcription level (CAGE-seq data from [40]). **c** Nucleosome dyad organization confirms the model prediction: promoters of active genes contain NDRs that are flanked by phased nucleosomes on both sides, while promoters of inactive genes contain disorganized nucleosomes and no NDR. The promoters of active genes (top ~ 40% of the genes) are accessible to DNaseI (**d**) (DNase-seq data from modENCODE [41], modENCODE_3324) and Tn5 transposase (**e**) (ATAC-seq data from [22]) and are bound by barrier complexes, such as the GAGA factor (**f**) (ChIP-seq data from [42]). **g**–**i** Heat maps of the nucleosome occupancy and the two fitted rates *k*_1_ and *k*_2_, indicating **g** the probability that a site is occupied by a nucleosome, **h** chromatin accessibility to MNase, as measured by the nucleosome release rate, and **i** decay rate

Previously, we have shown that nucleosome organization in *S. cerevisiae* can be predicted using a simple biophysical model of “barrier complexes” occupying promoters and steric hindrance between nucleosomes and the barrier complexes [10, 39]. Our model predicts that promoters that are occupied by such barrier complexes will be characterized by an NDR flanked by phased nucleosomes on both sides, while promoters that are not occupied by barrier complexes will not have an NDR and nucleosomes will be more disorganized (out-of-phase) (Fig. 10a). On the one hand, *S. cerevisiae* promoters are all relatively active and presumably occupied by general regulatory factors (such as Abf1, Rap1, and Reb1) and other transcription factors. Therefore, all yeast promoters are of the first type, characterized by NDRs and phased nucleosomes. On the other hand, *Drosophila* has multiple tissues, and each cell type has distinct sets of active genes and silent genes, which makes S2 cells an ideal setting for testing the prediction of our nucleosome positioning model (Fig. 10a).

To test this prediction, we sorted all *Drosophila* genes by their transcription level as measured by CAGE-seq (data from [40]) and aligned the genes at their TSS (Fig. 10b). Then, we plotted the normalized nucleosome dyad distribution, using the same alignment (Fig. 10c), which confirmed the prediction of our biophysical model. The active genes, containing barrier complexes required for gene activation at their promoters, have a distinct nucleosome organization from silent genes, as predicted by our model (Fig. 10a). While the positions of nucleosomes that are found on silent genes are determined by weak DNA sequence preferences, the positions of nucleosomes found near other strong DNA-binding proteins from promoters are determined by an interplay of steric exclusion by the barrier complexes, statistical positioning, and the action of chromatin remodelers [12]. To confirm that the barrier complexes are present in the promoters of the active genes, we analyzed DNase-seq data [41] and ATAC-seq data [22] (Fig. 10d, e) which are usually used to detect the active functional regions and the presence of regulatory factors. Moreover, we analyzed the distribution of GAGA factor, which is one of the most abundant DNA-binding proteins in *Drosophila*. As expected, the GAGA factor was detected at the promoters of genes characterized by NDRs and phased nucleosomes, suggesting that GAGA factor is one of the components of the barrier complexes in flies.

The fitted parameters of our kinetic model of chromatin digestion by MNase (nucleosome occupancy *O*, nucleosome release rate constant *k*_1_, nucleosome decay rate constant *k*_2_) confirmed that the most active genes have nucleosome-depleted promoters and the inactive genes have nucleosome-filled promoters (Fig. 10g). Also, the increased accessibility that we obtained for the gene promoters (Fig. 10h, i) correlate with the previous results obtained by DNase-seq and ATAC-seq (Fig. 10d, e).

Taken together, genomic data supports a statistical positioning model, in which barrier complexes occupying promoters of active genes phase nearby nucleosomes resulting in a stereotypical nucleosome organization of regular arrays of well-positioned nucleosomes flanking the nucleosome-depleted promoters.

### Nucleosome positions in S2 and Kc167 cells are very similar, but their occupancies are cell type-specific

To validate the current protocol and method of analyzing nucleosome positions and occupancy, we repeated the experiments in a second biological replicate and also in a different cell line (Kc167 cells). All the results were confirmed using the new sets of experiments. Moreover, the typical nucleosome positions that we identified in the two cell types showed a good agreement. In all experiments, we identified > 677,000 nucleosomes, and about 80% of the nucleosomes have similar positions in both cell types (shifted by 20 bp or less). When nucleosome positions identified in the same cell type (different biological replicates) were compared, about 93% of the nucleosome positions found in S2 cells were shifted by 20 bp or less, and about 87% of the positions were shifted by at most 10 bp. Interestingly, when we compared the nucleosome occupancies at the common positions that were identified in the two cell types, we obtained a Pearson correlation coefficient of only 0.36 (replicate 1) and 0.30 (replicate 2). These are substantially lower than the Pearson correlation between the occupancies estimated from different replicate experiments using the same cell type (> 0.78). This suggests that although the nucleosomes occupy about the same positions in S2 and Kc167 cells, the fractions of cells that contain a nucleosome at these positions (nucleosome occupancy) is very different in the two cell types.

## Discussion

It has been traditionally assumed that in MNase-seq experiments, the nucleosome fragment counts along the genome are proportional to the fraction of cells containing a nucleosome at the corresponding locus. More recently, it was observed that the nucleosome fragment counts obtained in different experiments depend on the specific digestion conditions and on the DNA sequence of each nucleosome [19, 21], but a rigorous theory that explains the variability observed in these experiments was still missing.

The *MACC* protocol [21], which separates nucleosomes into two distinct classes—accessible (characterized by a positive *MACC* score) and inaccessible (characterized by a negative *MACC* score)—partially addresses the issue of distinguishing nucleosome occupancy from DNA accessibility. However, this simple classification was limited to mononucleosomes recovered in a digestion series and could not account for nucleosome losses that occur with over-digestion. Indeed, we observed considerable heterogeneity between loci with respect to MNase sensitivity. Whether or not nucleosome losses seriously affected their conclusions could not be ascertained in the absence of a spike-in calibration standard, which is required for quantitative comparison of samples in genome-wide profiling experiments [43, 44]. Specifically, when digestion begins, all nucleosome counts are increasing with the level of digestion (more nucleosomes are released from chromatin), while in the latest stages of digestion, all nucleosome counts are decreasing with the level of digestion (more released nucleosomes are beginning to be destroyed by MNase) [45]. In other words, all nucleosomes can be characterized by a negative *MACC* score during the initial stages of digestion and by a positive *MACC* score during the final stages of digestion.

Here, we have provided a theoretical framework that explains the observed behavior of the nucleosome counts during the entire MNase digestion (from the start, when all nucleosomes are in chromatin, to the end, when all nucleosomes have been destroyed by MNase). We have developed a quantitative way of measuring the fraction of nucleosomes that are released from chromatin (q-MNase-seq), which allowed us to test and validate the predictions of the theoretical model.

## Conclusions

Using the newly developed protocol, we have found that some promoters are weakly protected by MNase-sensitive complexes, which are digested at a much higher rate compared to normal nucleosomes (one to two orders of magnitude difference between the corresponding *k*_1_ rates). Comparing the nucleosome release rates for nucleosomes originating from different regions of the genome, we found that DHSs are also more accessible to MNase, and nucleosomes from these loci are released almost twice as fast as the nucleosomes away from DHSs. Surprisingly, when we compared the euchromatin and heterochromatin regions, we could not detect major differences between the chromatin accessibility of these regions, as previously reported in human cells [46]. The nucleosome release rates for the chromatin regions annotated as heterochromatin and euchromatin [38] are comparable to each other, indicating that MNase can access nucleosomes in these regions at similar rates. This suggests that, although heterochromatin and euchromatin appear different when observed cytologically at low resolution, at the molecular level, MNase and other proteins can access heterochromatin regions at rates similar to those of accessing euchromatin. Our findings support the conclusions that have been drawn from nucleosome resolution imaging, in which the difference between heterochromatin and euchromatin is in the density of chromatin, but not in higher-resolution features, such as the average diameter of the chromatin fiber [47]. By developing a quantitative protocol for MNase-seq, we are thus able to take advantage of the high penetrability of MNase to distinguish nucleosome position and occupancy from higher-order chromatin properties such as density and compaction.

## Methods

### Biological materials

*Drosophila melanogaster* S2 cells were obtained from the Drosophila Genome Resource Center (Stock #181) and grown in HyClone SFX-Insect media supplemented with 18 mM l-glutamine. *D. melanogaster* Kc167 cells were obtained as a gift from Lucy Cherbas and grown in HyClone SFX-Insect media supplemented with 18 mM l-glutamine and 10% fetal bovine serum.

### MNase-seq

MNase-seq was performed on unfixed cells as described [32] with the following modifications: For each time point, ~ 2 million cells were suspended in 10 mM HEPES pH 7.4, 0.5 mM PMSF, and 0.5% NP40 on ice in a 166-μL volume. MNase was added at a concentration of 2.5 U per million cells. The mixture was warmed to 37 °C before the addition of 3.5 μL 100 mM CaCl_2_ (to 2 mM) to activate the MNase. Reactions were stopped by the addition of 170 μL of 2XSTOP (4.35 mL TM2, 340 μL 5 M NaCl, 200 μL 0.5 M EDTA, 100 μL 0.2 M EGTA, 25 μL RNase A, and 2 pg/mL yeast mononucleosomal spike-in DNA). After phenol-chloroform-isoamyl alcohol extraction and ethanol precipitation, the DNA pellet was dissolved in 50 μL 1 mM Tris-HCl pH 8, 0.1 mM EDTA and used directly for 1.5% agarose gel analysis and for making sequencing libraries as described [32]. For yeast spike-in DNA, spheroplast nuclei [48] were digested with MNase down to mostly mononucleosomes and extracted with phenol-chloroform-isoamyl alcohol as described [16].

### DNA sequencing and data processing

Paired-end sequencing (PE25x25) was performed on barcoded libraries using an Illumina Hi-seq 2500. Reads were aligned to the *D. melanogaster* reference genome *dm6* and *S. cerevisiae* reference genome *sacCer3*, using bowtie2 [49] with parameter --very-sensitive. Digestion levels and distributions of DNA fragments were analyzed using plot2DO (https://github.com/rchereji/plot2DO). Nucleosome counts (defined as the number of *Drosophila* DNA fragments with the length between 100 and 200 bp) and spike-in counts (number of *S. cerevisiae* DNA fragments with the length between 100 and 200 bp) were obtained using MATLAB (Bioinformatics toolbox). Raw genome-wide nucleosome count profiles were normalized by the corresponding spike-in counts, such that the resulting number of spike-ins was 10,000 for every sample. The typical locations of all well-positioned nucleosomes were detected using a custom MATLAB script, using the map consisting of all detected nucleosomes (from all 6 levels of digestion). For every detected nucleosome (~ 700,000), we used Eq. (2) to fit the distribution of the nucleosome counts as a function of digestion time and obtained the three parameters: nucleosome occupancy *O*, nucleosome release rate constant *k*_1_, and nucleosome decay rate constant *k*_2_. The non-linear curve fitting for all profiles was done in MATLAB, using the function lsqcurvefit. To visualize specific loci, igvtools was used to create tracks (tdf files) for viewing in the IGV browser [50]. Heat maps were plotted in MATLAB using the heatmap function (http://www.mathworks.com/matlabcentral/fileexchange/24253-customizable-heat-maps). Additional details of the biophysical model of chromatin digestion by MNase and data analysis are available in the Additional file 1.

## Supplementary information

Additional file 1: A quantitative investigation of MNase titrations. **Figure S1.** Dependence of the three species of nucleosomes on the digestion level. **Figure S2.** The predicted apparent nucleosome occupancy depends on the real occupancy O, and the two rates k1 and k2. **Figure S3.** The logarithm of the apparent nucleosome occupancy has the asymptotic behavior of −k2d for d ≫ 1/k2. **Figure S4.** Overview of a quantitative MNase-seq (q-MNase-seq) experiment. **Table S1.** The number of paired-end sequencing reads with the length between 100 bp and 200 bp. **Figure S5.** Simulation results. **Figure S6.** IGV snapshot of chromosome 2L: 13,789,750 - 13,794,300. **Figure S7.** Correlation between sets of parameters obtained after different normalizations of the nucleosome counts. (PDF 4086 kb)

Additional file 2: Review history. (DOCX 30 kb)

## Data Availability

Raw and processed data: Gene Expression Omnibus [51], RNA-seq data [21], ATAC-seq data [22], CAGE-seq data [40], DNase-seq data [41], and GAGA factor ChIP-seq data [42]. Source code (Custom MATLAB, R, and Bash scripts): GitHub [52] and Zenodo [53].
