# Supplementary material for: Quantitative MNase-seq accurately maps nucleosome occupancy levels
Source: Genome Biol. 2019 Sep 13;20:198. doi: 10.1186/s13059-019-1815-z (PMC6743174; doi:10.1186/s13059-019-1815-z)
Supplement: Supplementary file 1 — A quantitative investigation of MNase titrations. Figure S1. Dependence of the three species of nucleosomes on the digestion level. Figure S2. The predicted apparent nucleosome occupancy depends on the real occupancy O, and the two rates k1 and k2. Figure S3. The logarithm of the apparent nucleosome occupancy has the asymptotic behavior of −k2d for d ≫ 1/k2. Figure S4. Overview of a quantitative MNase-seq (q-MNase-seq) experiment. Table S1. The number of paired-end sequencing reads with the length between 100 bp and 200 bp. Figure S5. Simulation results. Figure S6. IGV snapshot of chromosome 2L: 13,789,750 - 13,794,300. Figure S7. Correlation between sets of parameters obtained after different normalizations of the nucleosome counts. (PDF 4086 kb) [file 13059_2019_1815_MOESM1_ESM.pdf]

Supplementary Information for

Quantitative MNase-seq accurately maps nucleosome occupancy levels

Răzvan V. Chereji, Terri D. Bryson, and Steven Henikoff

Contents

Kinetics of nucleosome release from chromatin 2
Mononucleosomal count versus nucleosome occupancy in MNase-seq experiments 4
Analysis of the nucleosome counts in series of MNase-seq experiments 5
Experimental design of q-MNase-seq 10
Paired-end sequence mapping 12
Data analysis 12
Detection of typical nucleosome positions 13
Nucleosome count normalization 14
Estimation of the errors 16
Other supplementary figures 17
Supplementary References 19

Kinetics of nucleosome release from chromatin

Here we analyze the kinetics of nucleosome release from a given genomic locus (e.g. the first (+1) nucleosome of a specific gene, or the nucleosome covering a specific transcription factor binding site). If we start the chromatin digestion experiment with a population of cells, then after a time t, some of the cells containing a nucleosome occupying this locus will release it from chromatin (if both of its linkers were cut by MNase), while other cells will still contain the corresponding nucleosome bound to longer chromatin fibers. Let's denote the relevant quantities as follows:

$[N](t)$−the concentration of mononucleosomes that were already released from chromatin and are still in the sample at time t (free nucleosomes);

$[B](t)$−the concentration of nucleosomes that are still bound to longer chromatin fibers at time t (bound nucleosomes);

$[E]$−the concentration of nuclease enzyme (MNase).

In order to release a nucleosome from chromatin, MNase must cleave the linkers on both sides of the nucleosome. After the nucleosome was released from chromatin, MNase uses its exo-nuclease activity and continues to trim the linker DNA until it reaches the nucleosome core particle. If the digestion reaction is not stopped, MNase will continue its exo-nuclease activity and invade the nucleosome, over-digesting and eventually destroying the nucleosome core particle. These two processes can be represented as a simple reaction chain,

$$B \xrightarrow{k_1} N \xrightarrow{k_2} \emptyset.$$

The first step of cutting the linker DNA and releasing the nucleosomes from chromatin is faster (rate constant k_1), while the second step of digesting the nucleosomal DNA by invading the nucleosomal core is slower (rate constant k_2). The rate of nucleosome release from a given locus depends on the chromatin accessibility of that locus: nucleosomes from more accessible regions are released faster, while nucleosomes from the inaccessible regions are released slower.

Assuming that DNA cleavage by MNase happens quickly after MNase binds to DNA (so the concentration of free enzyme $[E]$ remains approximately constant during the experiment), the concentrations of free and chromatin-bound nucleosomes satisfy the following reaction equations:

$$\frac{\mathrm{d}[B]}{\mathrm{d}t} = -k_1[B][E], \tag{1}$$

$$\frac{\mathrm{d}[N]}{\mathrm{d}t} = k_1[B][E] - k_2[N][E]. \tag{2}$$

Equation (1) has the solution,

$$[B](t) = [B]_0 e^{-k_1[E]t},$$

where $[B]_0 = [B](0)$ is the initial concentration of nucleosomes occupying a specific locus in a population of cells. Let's denote the concentration of cells that is used in the experiment by $[C]$, and the fraction of cells that has a nucleosome occupying the locus that we analyze by O, which is usually called the nucleosome occupancy [1]. Then, we have that $[B](0) = [C]O$, and the solution of Eq. (1) becomes

$$[B](t) = [C]Oe^{-k_1[E]t}.$$

Substituting this solution into Eq. (2), we obtain

$$\frac{\mathrm{d}[N]}{\mathrm{d}t} = k_1[C][E]Oe^{-k_1[E]t} - k_2[N][E].$$

The solution of this equation that satisfies the initial condition $[N](0) = 0$ is

$$[N](t) = [C]O\frac{k_1}{k_1 - k_2}\left(e^{-k_2[E]t} - e^{-k_1[E]t}\right). \tag{3}$$

Therefore, we obtained that after time t, the fraction of nucleosomes occupying a given position, which are still bound to chromatin is

$$f_B(t) = \frac{[B](t)}{[B](0)} = e^{-k_1[E]t},$$

the fraction of nucleosomes that were released from chromatin and were not yet destroyed by over-digestion is

$$f_N(t) = \frac{[N](t)}{[B](0)} = \frac{k_1}{k_1 - k_2}\left(e^{-k_2[E]t} - e^{-k_1[E]t}\right),$$

and the fraction of nucleosomes that were already destroyed by MNase is

$$f_\emptyset(t) = 1 - f_N(t) - f_B(t) = 1 - \frac{k_1}{k_1 - k_2}\left(e^{-k_2[E]t} - e^{-k_1[E]t}\right) - e^{-k_1[E]t}.$$

The product $[E]t$ represents a measure of the digestion level. In practice, the digestion level is controlled either by modifying the digestion time, t (digestion time course), or by modifying the concentration of enzyme, $[E]$ (MNase titration). As $[E]$ and t always appear as a product in the above Equations, for convenience, let's denote this measure of the digestion level by $d = [E]t$. Using this notation, we have that

$$f_B(d) = e^{-k_1 d}, \tag{4}$$

$$f_N(d) = \frac{k_1}{k_1 - k_2}\left(e^{-k_2 d} - e^{-k_1 d}\right), \tag{5}$$

$$f_\emptyset(d) = 1 - \frac{k_1}{k_1 - k_2}\left(e^{-k_2 d} - e^{-k_1 d}\right) - e^{-k_1 d}. \tag{6}$$

Supplementary Figure 1 shows the dependence of these three functions on the digestion level d: f_B – the fraction of chromatin-bound nucleosomes (blue line); f_N – the fraction of unbound nucleosomes that are still intact in the sample (red line); and $f_\emptyset$ – the fraction of nucleosomes that were destroyed by MNase during the course of digestion (yellow line).

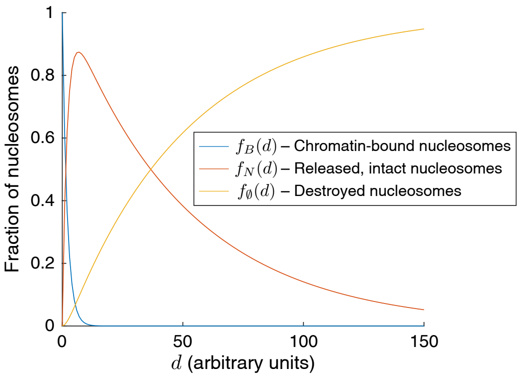

Fig. S1 | Dependence of the three species of nucleosomes on the digestion level, given by Eqs. (4)**,** (5)**, and** (6)**.** Function f_B represents the fraction of nucleosomes that are chromatin-bound and still not released as mononucleosomes (blue line); f_N represents the fraction of released nucleosomes that are still intact in the sample (red line); and $f_\emptyset$ represents the fraction of nucleosomes that were released from chromatin but have been already destroyed by MNase during the course of digestion (yellow line). The horizontal axis, d, represents a measure of the level of chromatin digestion, $d = [E]t$. Parameters used for predicting the corresponding fractions of nucleosomes: $k_1 = 0.2$, $k_2 = 0.02$.

Mononucleosomal count versus nucleosome occupancy in MNase-seq experiments

Equation (3) shows that the number of nucleosomes that were released from a genomic locus and remained intact at time t, depends on the total number of cells, the nucleosome occupancy at the given locus, O, but also on the level of digestion (affected by the concentration of MNase that was used in the experiment, $[E]$, and the time of digestion, t), and the two rate constants for nucleosome release and nucleosome decay, k_1 and k_2, respectively.

In an MNase-seq experiment, the level of digestion, $d = [E]t$, is fixed and the genome-wide distribution of the mononucleosomal reads is used to analyze nucleosome positions and nucleosome occupancy. When the level of digestion is fixed and the genome-wide nucleosome distribution is considered, this will be affected by the quantities O, k_1, and k_2, which may all vary along the genome. Equation (3) can be used to obtain the distribution of mononucleosomes at different genomic loci, x, as a function of the digestion level, d,

$$[N](x,d) = [C]O(x)\frac{k_1(x)}{k_1(x) - k_2(x)}\left(e^{-k_2(x)d} - e^{-k_1(x)d}\right). \tag{7}$$

In previous MNase-seq studies, it was generally assumed that the number of nucleosomes that were obtained from a genomic locus was a good measure of the actual nucleosome occupancy, i.e the fraction of cells containing a nucleosome at a given position. In other words, it was assumed that the mononucleosome count is directly proportional to the nucleosome occupancy. However, this "apparent" nucleosome occupancy obtained in MNase-seq experiments (i.e. the nucleosome

count) is clearly *not* directly proportional to the nucleosome occupancy, because there exists no constant α such that

$$[N](x) = \alpha O(x)$$

for all genomic loci x, no matter how one optimizes the digestion level d. As shown by Eq. (7), the apparent nucleosome occupancy also depends on other parameters ($k_1(x)$ and $k_2(x)$) that in general vary along the genome, due to differences in chromatin accessibility and DNA sequence.

In conclusion, the genome-wide nucleosome occupancy *cannot* be quantified by simply counting the number of mononucleosomal fragments that result from a single MNase digestion of chromatin, and the counts of mononucleosomes obtained from different genomic loci are not an accurate measure for the real nucleosome occupancy. A low number of mononucleosomal fragments observed at a given locus can indicate any of the following three scenarios:

(i) The nucleosome occupancy at this locus is low (this scenario was assumed to be true in previous MNase studies);

(ii) This locus is accessible to MNase and the nucleosome core particles occupying it in different cells have been already over-digested and destroyed before the reaction was stopped and the remaining intact nucleosomes have been collected;

(iii) This locus is less accessible to MNase and a more extensive digestion is necessary in order to release these nucleosomes from chromatin.

So how can we obtain a valid measure for the nucleosome occupancy, one that allows us to compare the nucleosome distribution at different regions along the genome? The following sections will provide a rigorous way of obtaining this, taking into account the combined effect of nucleosome occupancy and nucleosome accessibility to the nucleosome counts that are obtained in MNase-seq experiments.

Analysis of the nucleosome counts in series of MNase-seq experiments

Let's analyze in more detail the apparent nucleosome occupancy (i.e. the number of mononucleosomal fragments) that is obtained at a given genomic location x, in a series of experiments in which MNase is used to produce various levels of chromatin digestion. As we have seen before, the number of mononucleosomes that are found in the sample depends on the degree of digestion d, nucleosome occupancy O, and the two digestion rates k_1 and k_2, which depend on the nucleosome accessibility, and the DNA sequence of each nucleosome.

Supplementary Fig. 2 shows the effect of all three parameters (O, k_1 and k_2) on the predicted number of mononucleosomes that are obtained in a series of MNase-seq experiments when the digestion level is varied. The nucleosome occupancy O controls the overall height and area of the predicted nucleosome

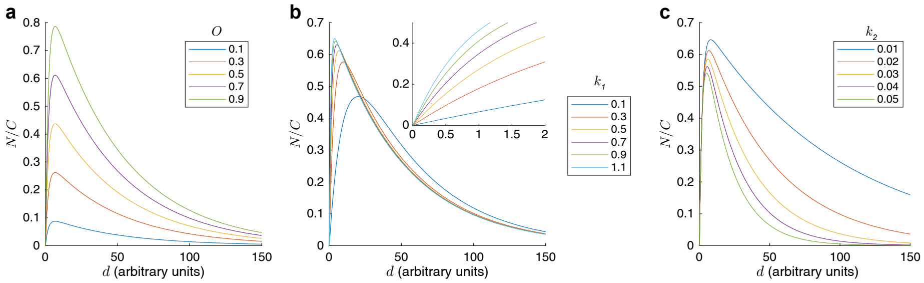

Fig. S2 | The predicted apparent nucleosome occupancy depends on the real occupancy O, and the two rates k_1 and k_2. (**a**) The dependence of N/C on the level of digestion, for different values of the nucleosome occupancy. For each value of the nucleosome occupancy, the apparent nucleosome occupancy obtained by counting the MNase-seq reads initially increases and later decreases back to 0, MNase starts to destroy the free nucleosomes. Other parameters used in these predictions: $k_1 = 0.5$; $k_2 = 0.02$. (**b**) The dependence of N/C on the level of digestion, for different values of k_1. The insert shows that the initial speed of releasing nucleosomes from chromatin is monotonically increasing with k_1. A higher initial rate of nucleosome release may indicate a higher chromatin accessibility. Other parameters used in these predictions: $O = 0.7$; $k_2 = 0.02$. (**c**) The dependence of N/C on the level of digestion, for different values of k_2. The rate k_2 controls the speed with which mononucleosomes are lost from the sample. Other parameters used in these predictions: $O = 0.7$; $k_1 = 0.5$.

counts as functions of the level of digestion d (Supplementary Fig. 2a). The rate k_1 controls the initial speed of accumulation of mononucleosomes in the early stages of the digestion (Supplementary Fig. 2b), and the rate k_2 controls the speed of nucleosome decay in the latest stages of the digestion (Supplementary Fig. 2c).

Note that uneven chromatin accessibility and different DNA sequences are likely to generate differences in the k_1 and k_2 rates along the genome. If any of the two rates, k_1 and k_2, are different or the level of digestion is different, then even loci with the same nuclosome occupancy can generate different nucleosome counts in MNase-seq experiments as predicted by Supplementary Fig. 2.

So, if the nucleosome count is not an accurate measure of the nucleosome occupancy, then how can we obtain one? And how can we also obtain the other two parameters that affect the number of mononucleosomes produced in a digestion series, k_1 and k_2?

The rate of nucleosome decay k_2 can be easily obtained by noticing that in the latest stages of the digestion (when $k_2 d \gg 1$) we have

$$
\begin{aligned}
N(d) &= CO\frac{k_1}{k_1 - k_2} e^{-k_2 d}\left[1 - \left(e^{-k_2 d}\right)^{\frac{k_1}{k_2}-1}\right] \\
&\approx CO\frac{k_1}{k_1 - k_2} e^{-k_2 d}, \text{ for } d \gg 1/k_2,
\end{aligned}
$$

so that

$$\log(N(d)) \approx \log\left(CO\frac{k_1}{k_1 - k_2}\right) - k_2 d,$$

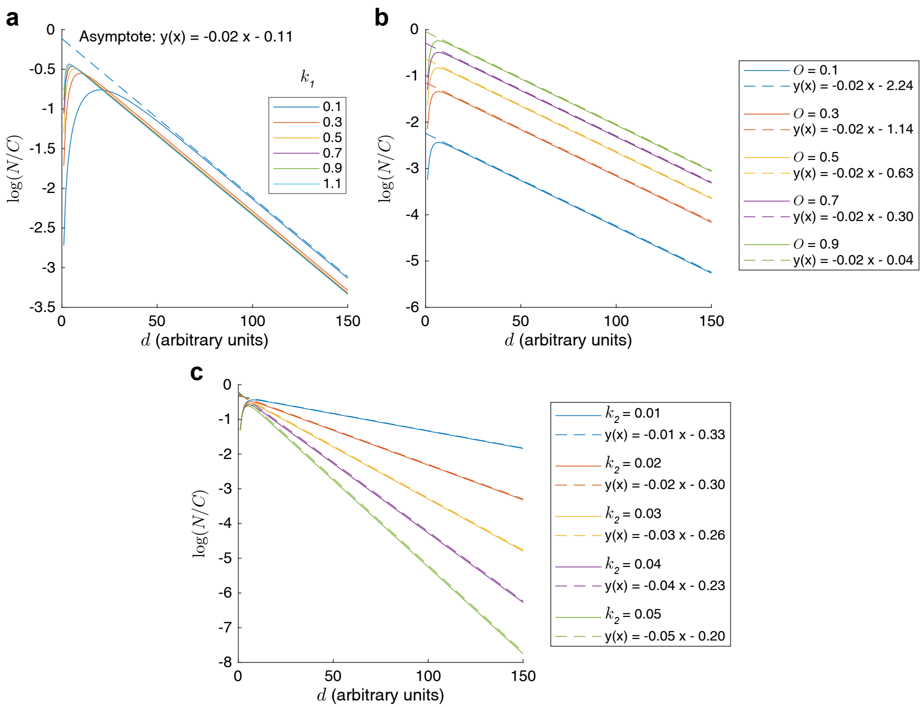

Fig. S3 | The logarithm of the apparent nucleosome occupancy has the asymptotic behavior of $-k_2 d$ for $d \gg 1/k_2$. Asymptotic behavior of $\log(N/C)$ for regions characterized by different k_1 rates (**a**), nucleosome occupancies (**b**), and k_2 rates (**c**). In the later stages of the digestion, $\log(N/C)$ becomes a straight line with the slope of$-k_2$, which can be easily obtained using a linear fit. Parameters used in the simulations: (**a**) $O = 0.7$; $k_2 = 0.02$; (**b**) $k_1 = 0.5$; $k_2 = 0.02$; (**c**) $O = 0.7$; $k_1 = 0.5$.

which decreases linearly with d. So, plotting $\log(N)$ as a function of d, we should see the same asymptotic behavior for all nucleosomes,

$$\log(N) \sim -k_2 d \text{ for } d \gg 1/k_2 \tag{8}$$

as shown in Supplementary Figure 3.

For the early stages of the digestion (when $d \to 0$), we have that

$$\begin{aligned} N(d) &= CO\frac{k_1}{k_1 - k_2}\left(e^{-k_2 d} - e^{-k_1 d}\right) \\ &\approx CO\frac{k_1}{k_1 - k_2}\left[(1 - k_2 d) - (1 - k_1 d)\right] = COk_1 d, \end{aligned} \tag{9}$$

and we see that, in the initial stages of digestion, the number of nucleosomes that are released from a given locus is proportional to the number of cells, C, the fraction of cells that contain a nucleosome at this locus, O, the rate of nucleosome release, k_1, and the level of digestion d. Therefore, initially,

increasing the level of digestion will always increase the number of nucleosomes that are released from chromatin, for all genomic locations.

We note that Mieczkowski et al. have previously claimed that nucleosomes can be characterized by a simple *MACC* score [2], arguing for the existence of an entire class of nucleosomes ("scenario 1" or "accessible chromatin") that are characterized by positive *MACC* scores. This indicates that during an MNase titration, the nucleosome counts (or fragment frequency, as denoted in [2]) corresponding to these nucleosomes are monotonically decreasing with the level of digestion. The prediction of our kinetic model is that all nucleosome counts are initially increasing with the level of digestion. Therefore, there exists no nucleosome for which the fragment frequency is monotonically decreasing even in the early stages of digestion, a regime that was overlooked by the authors of the previous study [2]. In other words, we question the validity of the proposed *MACC* scores, and we propose an improved model that gives the evolution of the nucleosome counts throughout the whole chromatin digestion process (Eq. (7)).

During an digestion time course, Equation (7) also allows us to compute the digestion level at which we'll obtain the maximum nucleosome count from a specific location x. Let's denote by $\widetilde{N}(x)$ the maximum number of reads that are obtained from a locus x. This corresponds to a digestion level $\widetilde{d}$, which is obtained by solving $\frac{\partial N}{\partial d} = 0$. From Eq. (7) we have that

$$\frac{\partial N}{\partial d} = CO\frac{k_1}{k_1 - k_2}\left(-k_2 e^{-k_2 d} + k_1 e^{-k_1 d}\right),$$

and therefore,

$$\left.\frac{\partial N}{\partial d}\right|_{d=\widetilde{d}} = 0 \Rightarrow k_1 e^{-k_1 \widetilde{d}} = k_2 e^{-k_2 \widetilde{d}},$$

and we obtain

$$\widetilde{d} = \frac{\log\left(\frac{k_1}{k_2}\right)}{k_1 - k_2}.$$

Moreover, the maximum number of nucleosomes that can be obtained from a genomic locus x is

$$\begin{aligned}\widetilde{N}(x) =& N(x, \widetilde{d}) \\ =& CO\frac{k_1}{k_1 - k_2}\left(e^{-\frac{k_2 \log\left(\frac{k_1}{k_2}\right)}{k_1 - k_2}} - e^{-\frac{k_1 \log\left(\frac{k_1}{k_2}\right)}{k_1 - k_2}}\right) \\ =& CO(x)\left(\frac{k_1(x)}{k_2(x)}\right)^{-\frac{k_2(x)}{k_1(x) - k_2(x)}}.\end{aligned}$$

If we compute the integral of $N(d)$ over all digestion levels (the area under the $N(d)$ curve), we obtain

$$\int_0^\infty N(x, d)\mathrm{d}d = CO(x)\frac{k_1(x)}{k_1(x) - k_2(x)}\left(\frac{1}{k_2(x)} - \frac{1}{k_1(x)}\right) = \frac{CO(x)}{k_2(x)}. \tag{10}$$

After we have estimated k_2 from the asymptotic behavior of $\log(N)$ for large d (Eq. (8)), we can now obtain a good measure of the true nucleosome occupancy, and of chromatin accessibility (measured by the rate of nucleosome release from chromatin, k_1). Equations (9), and (10) give us the slope of $N(d)$ for mild digestions and the area under the $N(d)$ curve:

$$\text{Initial slope}_{N(d)} = \left.\frac{\partial N}{\partial d}\right|_{d\to 0} = COk_1. \tag{11}$$

$$\text{Area}_{N(d)} = \int_0^\infty N(x,d)\mathrm{d}d = \frac{CO}{k_2}, \tag{12}$$

Using these results, one can easily obtain:

$$O = \frac{\text{Area}_{N(d)} \cdot k_2}{C} \tag{13}$$

$$k_1 = \frac{1}{k_2}\frac{\text{Initial slope}_{N(d)}}{\text{Area}_{N(d)}} \tag{14}$$

Using Eqs. (8), (13), and (14), we can estimate the parameters k_2, O, and k_1, respectively.

Alternatively, one can do a non-linear fit of the data using Equation (7), and find all three parameters for each site, $O(x), k_1(x)$ and $k_2(x)$, e.g. by using the `lsqcurvefit` function in MATLAB.

Unfortunately there is one more technical complication. For DNA sequencing, one needs to prepare a library that contains a sufficient amount of DNA. In order to obtain enough DNA, the original DNA is usually amplified by a number of PCR cycles. During the sequencing process only a fraction of the whole input DNA of each sample is sequenced, which depends on a variety of factors, such as the concentration of input DNA, the number of samples that are multiplexed in a sequencing lane, and the throughput/type of the sequencer. Since we want to detect quantitative differences between the number of mononucleosomes that were released after different levels of chromatin digestion, we need to be able to compare nucleosome counts among multiple samples. The current MNase-seq protocol does not allow a rigorous comparison between the nucleosome reads that are obtained in different experiments. In general, MNase-seq counts originating from different experiments are first normalized such that the total number of reads is assumed to be constant in all experiments. For our purpose, this normalization is not valid, as we expect that the total number of nucleosomes released from a population of cells will vary during different stages of the digestion. Equation (7) predicts that the total number of released nucleosomes will increase during the early stages of the digestion, and later it will change the trend and will start decreasing, in the latest stages of the digestion. Therefore, assuming that the total number of nucleosomes released at every digestion level is constant would compromise the results obtained using our theoretical framework.

Below, we present a new protocol for a quantitative MNase-seq (q-MNase-seq) experiment, which will allow us to use a proper normalization of the nu-

cleosome counts, and to compare the effective numbers of nucleosomes that are released from a population of cells, for each different level of digestion.

Experimental design of q-MNase-seq

In typical MNase-seq experiments (but also in other genome-wide assays, as ChIP-seq, ATAC-seq, DNase-seq, etc.), the total number of reads is usually normalized to a common library size (e.g. 1 million reads, to obtain all coverage profiles expressed in reads per million), to the length of the genome (to have all profiles expressed relative to the genome average, which is 1 read/bp in this case), or to another common number that is used for the normalization of all samples. The result of all these normalizations is that all samples will end up having the same total number of reads, which is thought to facilitate further comparisons among different samples. Unfortunately, many times this normalization procedure does not help, and actually makes the comparison among different samples impossible. We encounter one such scenario if we want to compare the transcription levels in different mutant cells by performing RNA polymerase II immunoprecipitation experiments. If in some mutant cells the overall level of transcription is increased or decreased compared to wild-type cells, then normalizing the ChIP-seq profiles to have the same total number of reads will effectively cancel the overall difference in the strength of the ChIP-seq signal. A similar problem arises if we want to compare the number of nucleosomes that are released in a time course experiment in which multiple samples are digested by MNase in various degrees. Again, it is expected that if we stop the reaction early or if we continue the chromatin digestion by MNase, then different numbers of nucleosomes will be released from chromatin: a more digested chromatin will result in more mononucleosomal particles and less nucleosomes remaining bound to the longer chromatin fibers.

To obtain a good measure of the real number of mononucleosoms that were released from chromatin and are available in the sample at each time during the course of digestion, we suggest the following q-MNase-seq procedure (Supplementary Fig. 4). We first split the cells in multiple samples, such that we start with approximately equal numbers of cells in each sample. These samples are then subjected to different degrees of chromatin digestion by MNase, either by doing an MNase titration or by using the same amount of MNase in all samples, but modifying the digestion times among the samples. The resulting undigested DNA fragments are collected from each sample, and an equal amount of spike-in DNA is added to all samples, to maintain the information about the relative amounts of nucleosomes that were found in each sample. We digest chromatin from *Drosophila* S2 cells or Kc167 cells, and use mononucleosomal DNA from *S. cerevisiae* as spike-in DNA. Next, sequencing libraries are created from the mix of fly and yeast DNA from each sample, and these are sequenced and mapped to both genomes.

This way, from each sample we obtain the total number of reads originating from *Drosophila* and the total number of reads originating from *S. cerevisiae.* Normalizing the read counts from each sample such that the total number of

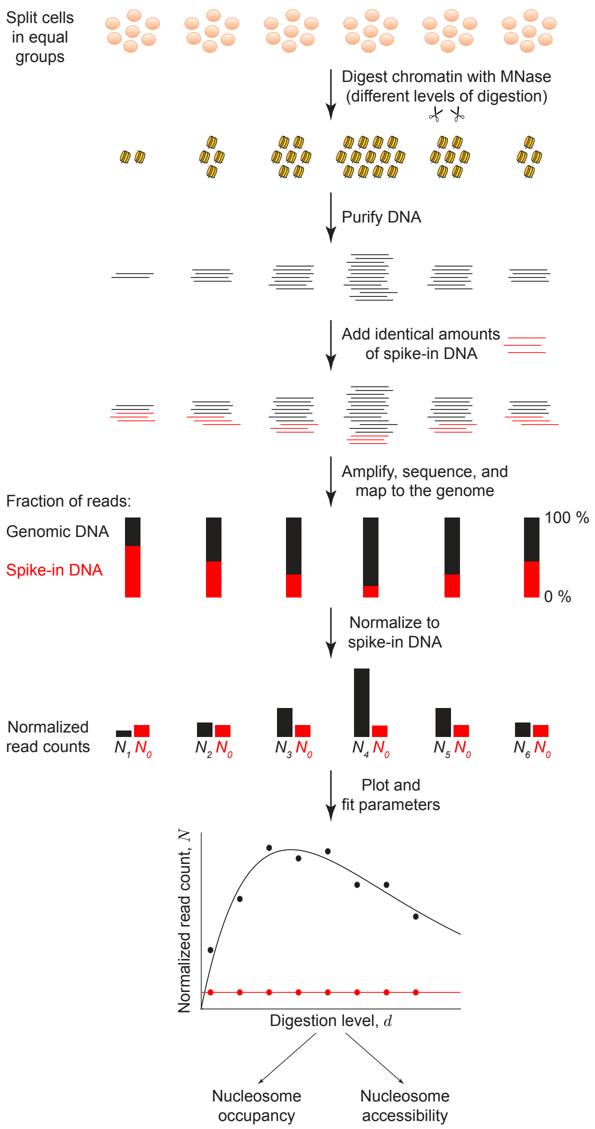

Fig. S4 | Overview of a quantitative MNase-seq (q-MNase-seq) experiment. *Drosophila* S2 cells are split into multiple groups, and digested with MNase, for various time intervals. Undigested DNA is purified, then equal amounts of spike-ins (yeast DNA) are added to each sample. After DNA sequencing and mapping to both genomes, we normalize the read counts from *Drosophila* by the amount of spike-in DNA. For each locus of interest, we fit the normalized nucleosome counts using Eq. (7), and we estimate the nucleosome occupancy and the rate of nucleosome release from the respective locus, which can be used as a measure of nucleosome accessibility.

reads from yeast is constant among all samples, we obtain a good measure of the initial number of nucleosomes that were released from chromatin at each

stage of the digestion, which can be plotted as a function of the digestion level (Supplementary Fig. 4). Obtaining a similar plot for every genomic locus allows us to fit the data using Eq. (7) and to obtain the following parameters: nucleosome occupancy, nucleosome accessibility, and nucleosome decay rate.

Paired-end sequence mapping

To align the sequencing reads to the *D. melanogaster* genome, we used `bowtie2` (version 2.3.4.1), `samtools` (version 1.9), the UCSC *dm6* reference genome downloaded from iGenomes - Illumina (`https://support.illumina.com/sequencing/sequencing_software/igenome.html`), and the following commands:

```
bowtie2 -X 1000 --very-sensitive -p 30 \
        -x <dm6_index> \
        -1 <dataset>_R1.fastq.gz \
        -2 <dataset>_R2.fastq.gz \
        2> <dataset>.dm6_alignment_statistics.log | \
samtools view -bS -f 0x2 -F 0x300 - | \
samtools sort -T tmp_<dataset> -o <dataset>.dm6.bam -
samtools index <dataset>.dm6.bam
```

Similarly, to align the sequencing reads to the *S. cerevisiae* genome and to obtain the spike-ins corresponding to each sample, we used the UCSC *sacCer3* reference genome and the following commands:

```
bowtie2 -X 500 --very-sensitive -p 30 \
        -x <sacCer3_index> \
        -1 <dataset>_R1.fastq.gz \
        -2 <dataset>_R2.fastq.gz \
        2> <dataset>.spikeins_statistics.log | \
samtools view -bS -f 0x2 -F 0x300 - | \
samtools sort -T tmp_<dataset> -o <dataset>.Scer_spikeins.bam -
samtools index <dataset>.Scer_spikeins.bam
```

The above commands will use the raw sequencing data from the FASTQ files, and align the corresponding DNA fragments to the reference genomes (*dm6* and *sacCer3*), and output the alignment data into BAM-formatted files.

Data analysis

To read the aligned data from the BAM files and for data analysis we used custom MATLAB and R scripts, provided at `https://github.com/rchereji/qMNase-seq`, but there are multiple options for other programming languages, as well. Briefly, the workflow can be described as follows. After read alignment using `bowtie2`, we obtained a set of BAM files containing the genomic locations of all paired-end reads that were sequenced and mapped to the reference genome. We examined the length distribution of the undigested fragments using the

function `get_DNA_fragment_lengths.m`. We imported the raw dyad counts using the function `get_raw_dyads.m` or `get_raw_dyads_and_occupancy.m`, which also computes the genome-wide coverage map (i.e. the raw nucleosome coverage/occupancy profile). To compute the amounts of spike-ins corresponding to each sample we used the function `count_Scer_spikeins.m`. To compare multiple profiles corresponding to different digestion levels, we must normalize the raw profiles to the corresponding spike-ins. This was done using the script `Step_04_1_Normalize_all_profiles_by_spikeins.m`. To visualize the normalized profiles in IGV, we used the function `write_profile_to_WIG_file.m` to generate WIG files, the bash script `Step_13_2_Convert_files_WIG_to_BigWig.sh` to generate BW (BigWig) files, and the script `Step_13_3_Convert_files_BigWig_to_TDF.sh` to generate TDF files (the preferred format for the IGV browser). We have noticed that some genomic regions gave a high number of reads compared to the rest of the corresponding chromosomes. This could result from the poor annotations of the corresponding regions (e.g. regions of DNA that are repeated multiple times in our S2/Kc167 cells but are annotated only once in the reference genome, copy number variations, etc.). To account for these local variations along the chromosomes, we computed a local normalization factor which represents the average sequencing depth in 2-kb windows relative to the chromosome average. This was computed using the script `Step_04_2_Get_local_normalization_factor_*.m`. Next, we detected the typical nucleosome positions of all well-positioned nucleosomes in S2 and Kc167 cells, as described in the following section.

Detection of typical nucleosome positions

Nucleosomes can occupy slightly different positions in different cells. If a nucleosome occupies about the same position in a population of cells, we call this a well positioned nucleosome. Nucleosomes that occupy a wide range of positions in a population of cells are called poorly positioned or "fuzzy nucleosomes". In practice, for each well positioned nucleosome, we observe a tight cluster of dyad positions, while for poorly positioned nucleosomes we observe a wide cluster of dyad positions, and a poor separation between the clusters of reads corresponding to neighboring nucleosomes.

To detect the typical positions of the well positioned nucleosomes along the whole genome, we used the following algorithm. We initialized equally spaced overlapping search widows with the width of 147 bp and their centers separated by 50 bp, which spanned all chromosomes. For each window, centered at position i, we computed the median coordinate of all dyads that were detected between positions $i - 73$ and $i + 73$], and then we updated the corresponding window center with the median position of the corresponding nucleosome dyad cluster. We repeated this window sliding procedure until the median position of the nucleosome dyad cluster centered at i became equal to $i \pm 1$, and no additional sliding was necessary. This search procedure was repeated for all starting points. At the end of this search algorithm, multiple windows could converge to the same positions, and we eliminated the duplicate positions. Finally, we inspected each

typical nucleosome position that we detected, and eliminated the peak calls for which the number of reads was bellow a threshold (its average occupancy at the central 101 bp is less that 20% of the genome average). We also eliminated the peak calls for which the average occupancy over the linkers (measured within two 10 bp intervals adjacent to the 147 bp nucleosome core) was comparable to the average occupancy over the central 101 bp – this situation corresponds to "fuzzy nucleosomes" for which a position cannot be well defined.

The algorithm described above was implemented in MATLAB, and is available on GitHub in the script `Step_05_1_Get_typical_nucleosome_positions_*.m`.

Nucleosome count normalization

In each q-MNase-seq experiment we digested chromatin from *D. melanogaster* cells, and we used spike-ins obtained from yeast digested chromatin. We studied a wide range of chromatin digestion levels, from a very weak digestion (1 min of MNase digestion) to a very extensive digestion (60 min of MNase digestion). We obtained various lengths of undigested DNA, from long fragments (∼200 bp) corresponding to nucleosomes attached to flanking linker DNA, to short fragments (∼100 bp) corresponding to overdigested nucleosomes, which have lost their last few helical twists of DNA, due to cleavages by MNase at the ends of the nucleosomal DNA, facilitated by spontaneous nucleosome unwrapping [3]. To account for all nucleosomes that were released from chromatin at any given stage of digestion, in each sample we counted all DNA fragments with the length between 100 bp and 200 bp, and the resulted number of reads are shown in the table below. The same size range was used to count the spike-ins in each experiment.

Table S1: The number of paired-end sequencing reads with the length between 100 bp and 200 bp.

**Digestion time**	**S2 cells, exp. 1** ($\times 10^6$)	**S2 cells, exp. 2** ($\times 10^6$)	**Kc167 cells, exp. 1** ($\times 10^6$)
1 min	71	21	18
2 min	58	18	21
5 min	59	18	15
15 min	51	15	12
40 min	40	20	27
60 min	38	18	20

To be able to to compare the nucleosome counts obtained at different levels of digestion, we normalized all profiles to a common number of 10,000 spike-ins, using the script `Step_04_1_Normalize_all_profiles_by_spikeins.m`. After this normalization, we could easily compare the spike-in normalized nucleosome counts obtained from different levels of digestion and from different

genomic regions. Since different levels of digestion result in different lengths of the protected DNA fragments (longer undigested footprints in the less digested samples), all nucleosomal fragments (100 bp $\leq$ L $\leq$ 200 bp) were symmetrically trimmed or extended to the normal nucleosome core particle size of 147 bp, using the function `extend_dyads_to_fixed_footprint.m`. The resulted homogeneous 147-bp fragments were stacked to obtain the normalized coverage profiles. For illustration purposes (to better distinguish neighboring nucleosomes in IGV plots), we also constructed nucleosome coverage profiles in which the reads were symmetrically trimmed to 101 bp from the original 147 bp, in order to emphasize the linker DNA, using the same function `extend_dyads_to_fixed_footprint.m`. These coverage profiles were only used to illustrate the regular arrays of nucleosomes and to detect the typical nucleosome positions (script `Step_05_1_Get_typical_nucleosome_positions_*.m`), but not to quantify the level of nucleosome occupancy at a given location.

Any sequencing experiment involves multiple steps. First, to generate a DNA library, sequencing adapters are ligated to the original DNA fragments. These adapters contain sequences complimentary to the flow cell oligos, and will attach the DNA fragments to the surface of the Illumina flow cell. Adapter dimers can easily bind and occupy space on the flow cell without generating any useful data, so adaptor dimers and other library preparation artifacts must be removed from the library. The next steps involve cluster generation by clonal amplification of the library, and reading the DNA fragments (sequencing by synthesis). Since each step has a finite efficiency, and we can only obtain the number of DNA fragments that successfully passed through all the previous steps, we do not have precise information about the DNA fragments that were not successfully sequenced. In other words, we only obtain the number of nucleosomal DNA fragments that were captured and sequenced by the sequencer, and not the total number of nucleosomes that were present in the sample, or the exact number of cells that released these nucleosomes in the sample. We can only assume that the number of reads that are obtained from the sequencer for each genomic locus is directly proportional to the number of nucleosomes that were originally present in the sample. The constant of proportionality is unfortunately unknown, as it depends on the efficiency of all steps that are involved in the sequencing experiment. Fortunately, the only effect of a multiplicative proportionality factor added to the apparent nucleosome occupancy, $[N]/[C]$ (Eq. (7)), is to rescale the nucleosome occupancy O, and the other fitted parameters will remain the same, when fitting a rescaled profile of the nucleosome counts.

When we fitted the nucleosome counts for all *Drosophila* nucleosomes to obtain the three parameters, O, k_1, and k_2, we confirmed that the effects of the rescaling factor that converts the nucleosome count N into aparent nucleosome occupancy N/C are negligible. Supplementary Figure 7 shows the Pearson correlation coefficients between the three sets of parameters that were obtained using the function `Step_08_1_Fit_parameters_*.m` and multiple rescaling factors that we tested. All correlation coefficients were extremely close to 1 (>0.9995); the k_1 and k_2 parameters were essentially identical in all cases that we tested,

while the O parameters were rescaled directly proportional to the normalization factor that was used in each case. The genome-wide average nucleosome occupancy ($< O >$) can be easily estimated from the average nucleosome repeat length ($< NRL >$) obtained by electrophoresis of MNase digestion products, $< O >= 147/ < NRL >$, or even from a rough estimate using the theoretical prediction of the expected filling density (or average occupancy) in a random filling/“parking” process (known as Rényi’s parking constant, $m = 0.747\ldots$ [4]). Therefore, one can easily rescale the nucleosome occupancy values obtained by fitting the nucleosome counts (using any rescaling factor for the apparent nucleosome occupancy), such that genome-wide average occupancy agrees with the one obtained from gel electrophoresis.

We analyzed the bands corresponding to Sample 3 from the gel shown in Figure 2 using GelAnalyzer (`http://www.gelanalyzer.com/`), and we obtained an estimation of the average NRL of 205 bp (standard error of the estimate: 4 bp). Using this value of $< NRL >$, we estimated that the genome-wide average nucleosome occupancy is $147/205 = 0.717$. This estimation was used to scale the fitted values for O, and to plot the genome-wide nucleosome distribution in Figure 10.

Estimation of the errors

To study the robustness of our fitting procedure we performed the following experiment. We generated 100,000 curves representing the fraction of nucleosomes that are released from chromatin from different loci and at different levels of digestion. Then, we considered 5, 10, or 15 different levels of digestion and we assumed that the nucleosome counts obtained from the sequencing experiments have a 10% accuracy. Therefore, for every true value of the nucleosome count given by Eq. (3), we added a noise term (taken from a uniform distribution) of up to ±10% of the real count. An example of the real fraction of nucleosomes released as a function digestion level and 5/10/15 noisy data points is shown in Supplementary Fig. 5a using a black line and colored circles (5 blue circles, 10 green circles, 15 red circles). Then, considering that a real experiment would measure these noisy values, we used Eq. (3) to fit these data points, using the `lsqcurvefit` nonlinear least-squares solver from MATLAB. The relative errors for all the fitted parameters are summarized in Supplementary Fig. 5b. Using only five data points, the median relative errors were 5.55% (O), 8.97% (k_1), and 9.61% (k_2), which further decreased by increasing the number of data points used for the fit.

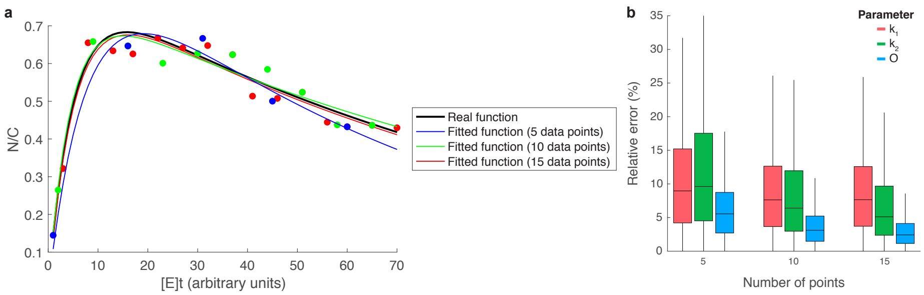

Fig. S5 | Simulation results. (**a**) Fitted curves obtained using five, ten, and fifteen data points. The real N/C function was computed using Eq. (3) (black line), and random values for O, k_1, and k_2. Five, ten, or fifteen random points were selected, and random noise of up to $\pm 10\%$ was added to the real values. Noisy data are shown with colored circles. The noisy data were fitted using Eq. (3), and the fits are shown using the red, green, and blue lines. (**b**) Summary of the relative errors obtained in 100,000 fit simulations, using random values for O, k_1, and k_2.

Other supplementary figures

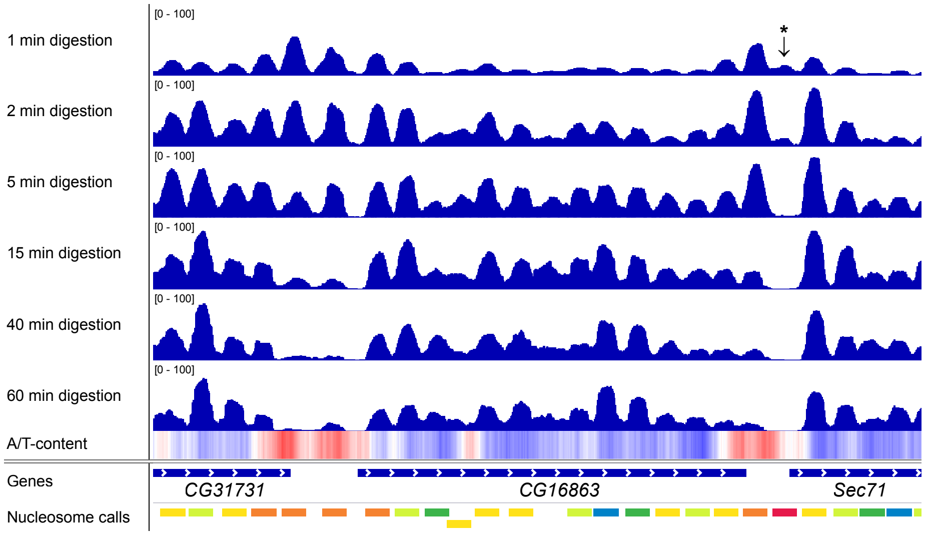

Fig. S6 | IGV snapshot of chromosome 2L: 13,789,750 - 13,794,300. Nucleosomes are digested at different rates by MNase depending on the DNA sequence of each nucleosome. Promoters and transcription terminators are usually A/T-rich regions, and the corresponding nucleosomes are released faster from chromatin compared to the ones orig-inating from G/C-rich regions. The A/T-content of the presented region is indicated as an heat map. White indicates the genome-wide average A/T-content in *Drosophila*, ~58%, red indicates A/T-rich regions and blue indicates G/C-rich regions. The rectangles in the bottom track indicate the nucleosome calls, and are colored as in Figure 3. The peak marked by an asterisk may indicate an MNase-sensitive complex that does not contain histones [5] and offers only a reduced protection against MNase.

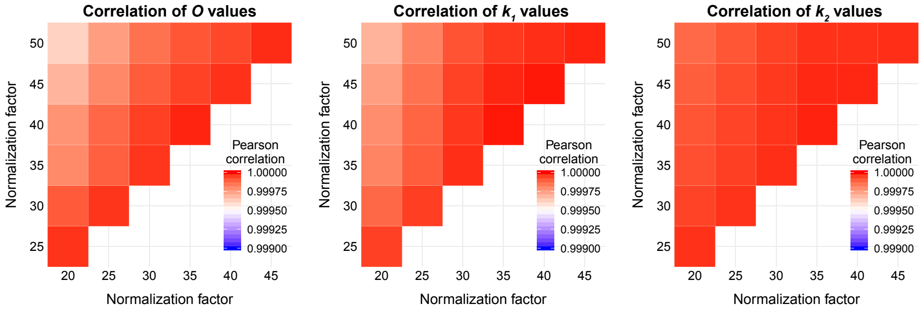

Fig. S7 | Correlation between sets of parameters obtained after different normalizations of the nucleosome counts. Pearson correlations obtained for the sets of parameters obtained after different normalizations of the nucleosome counts are greater than 0.9995 in all cases.

Supplementary References

[1] Chereji, R. V. & Clark, D. J. Major determinants of nucleosome positioning. *Biophys. J.* **114**, 2279–2289 (2018).

[2] Mieczkowski, J. *et al.* MNase titration reveals differences between nucleosome occupancy and chromatin accessibility. *Nat. Commun.* **7**, 11485 (2016).

[3] Chereji, R. V. & Morozov, A. V. Ubiquitous nucleosome crowding in the yeast genome. *Proc. Natl. Acad. Sci. USA* **111**, 5236–5241 (2014).

[4] Rényi, A. On a one-dimensional problem concerning random space-filling problem. *Publ. Math. Inst. Hungar. Acad. Sci* **3**, 109–127 (1958).

[5] Chereji, R. V., Ocampo, J. & Clark, D. J. MNase-sensitive complexes in yeast: nucleosomes and non-histone barriers. *Mol. Cell* **65**, 565–577 (2017).
