# Supplementary material for: Quantitative MNase-seq accurately maps nucleosome occupancy levels
Source: Genome Biol. 2019 Sep 13;20:198. doi: 10.1186/s13059-019-1815-z (PMC6743174; doi:10.1186/s13059-019-1815-z)
Supplement: Supplementary file 2 — Review history. (DOCX 30 kb) [file 13059_2019_1815_MOESM2_ESM.docx]

Review History

**First round of review**

**Reviewer 1**

**Were you able to assess all statistics in the manuscript, including the appropriateness of statistical tests used? Yes:** The mathematical modeling seems sound, but is beyond my expertise to access in detail.

**Were you able to directly test the methods? No**

**Comments to author:**

In this manuscript, Chereji and colleagues combined serial MNase digestion of chromatin with spike-in controls and mathematical modelling to improve the measurement of nucleosome occupancy on a genome wide scale. The authors very prominently demonstrate the effect of AT-content on MNase digestion and identified subsets of nucleosomes with different digestion kinetics. In particular, they identify a small subset of nucleosomes that are "hypersensitive" to MNase digestion a likely represents chromatin remodeling complexes associated with transcription factor binding sites at promoter regions.

This study consists of a thorough set of experiments, which are mostly well analyzed. MNase-seq is far from perfect, yet the easiest to use approach to investigate the nucleosome landscape, hence realization of its pitfalls and improvements brought by this study are very welcome. I however have few major and minor points to consider.

Main points:
1) While this study very elegantly demonstrates some facts that are sort of known (e.g. AT-preference of MNase, presence of hypersensitivity around TF binding sites) I recon the main value of this manuscript is the improved quantitative nature of the MNase-seq approach. To this end, I was however disappointed that the manuscript does not provide clear recommendation and/or tools how future studies should make use of the knowledge generated in this study. E.g. what would be a minimal number of digestion steps required to accurately predict nucleosome occupancy? Could we use the size and/or AT content of the MNase digested fragments as a predictor for of over-digestion (as suggested by an earlier study of the Henikoff lab) and reduce the number of digestion conditions? Clear answer to these questions would be essential, while a software predicting nucleosome occupancy would be nice addition.
2) It appears that this study is based on a single digestion series from a single drosophila cell line. In my opinion, it would be essential to repeat it at least one or two more times to be able to estimate whether variation observed in nucleosome occupancy are robust features of the different nucleosomes or simple variation between nucleosomes in this specific experiment. Comparing occupancy levels in two different cell lines/states would only be a nice addition, but not essential.
3) It appears that the authors focused their analysis on "highly positioned" nucleosomes. While this was probably the most straightforward to do, I was wondering how this approach improves the measurement of nucleosome occupancy for less positioned nucleosomes. More importantly, how this selection might have influenced the conclusions drawn. In particular, I am not sure whether the proportion of positioned nucleosomes are the same in the different chromatin domains (Figure 9c) and if not is it safe to conclude that heterochromatin contains equally accessible nucleosomes as compared to other domains or this is based on a specific subset of positioned nucleosomes located in this domain. 

Minor points:
-     Figure 8 suggests that regions with vastly different occupancy values exist (10-80%), but it is not indicated what proposition of the genome/nucleosomes falls under these different categories and no further analysis presented to explore the reason for these massive differences. 
-     Figure 6 suggest that digestion kinetics of neighboring nucleosomes are not entirely independent. If this is indeed the case, this would be worthwhile mentioning and perhaps discussing the effect of this on calculation of nucleosome occupancy.

**Reviewer 2**

**Were you able to assess all statistics in the manuscript, including the appropriateness of statistical tests used? Yes:** The statistical tests are appropriate

**Were you able to directly test the methods? No**

**Comments to author:**

The authors provide a nice study aiming to offer a quantitative description of MNase-seq experiments. This is an uneasy but important task, and these results will be interesting to a number of scientists working in this area. The article is in general well-written and appropriate for Genome Biology. My suggestions are provided below in the order of the manuscript flow:

1) Abstract: "Surprisingly, we observed no overall differences in nucleosome occupancies between Drosophila euchromatin and heterochromatin…"
- I suggest to cite in the text in this respect a recent paper that also did not find differences in MNase accessibility between eu- and heterchromatin: https://www.ncbi.nlm.nih.gov/pubmed/30496478, as well as a paper from 1979 that compared the kinetics of MNase digestion in the intact chromatin vs in solution: https://www.ncbi.nlm.nih.gov/pubmed/489546.

2) Abstract: "…which implies that heterochromatic compaction does not reduce accessibility of linker DNA"
- I would say that this statement should refer only to *MNase accessibility*, because some other proteins may still have different accessibility to linker DNA.

3) Introductions: "If the number of nucleosomal fragments obtained
from a given locus increases with the MNase concentration, then this locus is… called "inaccessible chromatin". If the number of nucleosomal fragments decreases with the MNase concentration… then this locus is … labeled as "accessible chromatin"

- This interpretation is based on certain strong assumptions. Another way of looking at MNase-seq titration with different digestion levels that is less dependent on assumptions is to separate nucleosomes into "stable" and "labile" (MNase-sensitive) as was done previously: https://www.ncbi.nlm.nih.gov/pubmed/24812327.

4) Results (pages 4 and 5): The computational method suggested here assumes that all MNase-seq digestion events across the genome are governed by the same biochemical equilibrium. It may be a proper assumption, but such assumption (and its limitations) needs to be written explicitly. For example, consider two locations X and Y. In location X there is a protein A sitting on the DNA and the DNA accessibility is determined by the kinetics of protein A dissociation. In location Y there is protein B sitting on the DNA that has a different dissociation kinetics. Similarly, some regions near the nucleosome dyad become accessible to MNase due to "nucleosome breathing" and this kinetics can be different in locations X and Y across the genome.

5) Equation 2: k1 and k2 are not defined in the text (although a definition appears in the legend of Figure 1). BTW, after noticing that k1 and k2 are defined as the functions of the location along the genome X in the legend of Figure 1, it seems that Equation 2 may need to be re-formulated by using explicitly the genomic coordinate X. It is also important to make the occupancy O(x) part of the equations in the main text.

6) Letter "N" is used to denote nucleosome occupancy in Equation 1. Is it the same as letter "O" in equation 2? Is the meaning of variable "N" used in Equation 2 different from the meaning of variable "N" in Equation 1? If so, it needs to be explained. 

7) Looking through the notation explained in the Supplementary Materials it seems that N(t) and B(t) are defined there as concentrations, whereas N is mentioned as the number of nucleosomes or as the nucleosome occupancy in the main text. It is important to unify the notation with respect to all variables in all places in the main text and Supplementary Materials.

8) Results page 7: "we add equal amounts of spike-in DNA (S. cerevisiae  mononucleosomal DNA obtained by a traditional MNase-seq method).

- How were these mononucleosomes extracted from the MNase preparation? This information needs to be added to the methods section. 

9) Results page 7: "Then, we normalize the counts of fly mononucleosomes by the amount of spike-in DNA from each sample"

- How exactly was the normalisation performed? What are the assumptions here?

10) Figure 8: Please add the best fit parameters for k1 and k2 to each panel.

11) Figure 9: It would be informative to show also the values of k2 and the average nucleosome occupancy for each case.

**Reviewer 3**

**Were you able to assess all statistics in the manuscript, including the appropriateness of statistical tests used? Yes:** The statistics is sound and solid.

**Were you able to directly test the methods? Yes**

**Comments to author:**

Review of the manuscript submitted by Chereji et. al, with the title ´Quantitative MNase-seq accurately maps nucleosome occupancy levels`.

The authors submitted a very good protocol to quantitatively analyse nucleosome occupancy. As chromatin of different genomic regions exhibits different MNase sensitivity and accessibility, nucleosomes are released at different rates. The authors provide a model and equation to quantify these effects, taking into account that nucleosome cores released early are prone to their disruption by hydrolysis of the nucleosomal DNA. They used Drosophila S2 cells to prepare  a time course of MNase hydrolisation, in order to test the proposed model. 
MNase is a widely used tool that exhibits a high preference for the AT-content of DNA, thereby preferentially hydrolysing AT-rich linker DNAs and releasing nucleosomes reconstituted on AT-rich genomic regions first. In turn these AT-rich nucleosomes could be also more susceptible to hydrolysis by MNase and being the first ones to be destroyed. 
The authors can convincingly show the early release of nucleosomes at AT-rich DNA sequences and observe  the subsequent elimination of these nucleosomal particles out of the pool. Increasing hydrolysis time results in nucleosomal and sub-nucleosome sized protected DNA with decreasing AT-content. These effects are not new, but the quantitative description of these effects has its merit.
The addition of the spike-in DNA to normalise the samples is essential for quantitative assessment and the color coding of the called nucleosomes according to their time point of maximal intensity is very appealing. The protocol should serve as a best practice example to plan and analyse MNase-seq data. 
However, as MNase-seq requires deep sequencing of the nucleosomal DNA, the preparation of six libraries and sequencing reactions would make this kind of experiments very expensive for large genomes. As the authors want to establish a general MNase-seq pipeline, they should address the question of how many time points are essential to reveal qualitative different nucleosome species, and/or their association with epigenetic marks/DNA sequence features/regulatory elements/etc.. Would the first and last time point of MNase hydrolysis be sufficient to obtain the same results, or are three time points sufficient?
I think that with the rigorous analysis of the MNase-seq data the authors provide an excellent protocol. However, besides this, the manuscript provides only incremental new insights to chromatin organisation. The finding that the MNase accessibility is similar for euchromatin and heterochromatin was previously published for human cells by Schwartz and Längst in Nucleic Acids Research (2019). Surprisingly the authors do not cite or discuss this manuscript. The methods are similar, however, Schwartz and colleagues use only two time points of MNase hydrolysis that are sufficient to draw the same conclusion. For this I think it is important to address the minimal number of digestion points, as described above.
Having this quantitative evaluation of nucleosome occupancy. Is it possible to normalise a genome for the AT-content and to provide a measure for an optimal degree of MNase digestion to the experimentalists? In order to have a golden standard for future MNase-seq experiments, this recommendation would be highly valuable for chromatin structure analysis.

**Authors Response**

**Point-by-point responses to the reviewers’ comments:**

Reviewer #1: 
In this manuscript, Chereji and colleagues combined serial MNase digestion of chromatin with spike-in controls and mathematical modelling to improve the measurement of nucleosome occupancy on a genome wide scale. The authors very prominently demonstrate the effect of AT-content on MNase digestion and identified subsets of nucleosomes with different digestion kinetics. In particular, they identify a small subset of nucleosomes that are "hypersensitive" to MNase digestion a likely represents chromatin remodeling complexes associated with transcription factor binding sites at promoter regions. 

This study consists of a thorough set of experiments, which are mostly well analyzed. MNase-seq is far from perfect, yet the easiest to use approach to investigate the nucleosome landscape, hence realization of its pitfalls and improvements brought by this study are very welcome. I however have few major and minor points to consider. 

Main points: 
1) While this study very elegantly demonstrates some facts that are sort of known (e.g. AT-preference of MNase, presence of hypersensitivity around TF binding sites) I recon the main value of this manuscript is the improved quantitative nature of the MNase-seq approach. To this end, I was however disappointed that the manuscript does not provide clear recommendation and/or tools how future studies should make use of the knowledge generated in this study. E.g. what would be a minimal number of digestion steps required to accurately predict nucleosome occupancy? Could we use the size and/or AT content of the MNase digested fragments as a predictor for of over-digestion (as suggested by an earlier study of the Henikoff lab) and reduce the number of digestion conditions? Clear answer to these questions would be essential, while a software predicting nucleosome occupancy would be nice addition. 

*To study the kinetics of nucleosome release from chromatin, one needs to estimate three parameters for each nucleosome: its occupancy, O, the rate of nucleosome release, k_1, and the rate of nucleosome decay due to overdigestion by MNase, k_2. Therefore, in principle, deep sequencing of three levels of digestion would be enough to estimate these three parameters for each nucleosome. Unfortunately, all data points are affected by some experimental error/noise, and using only three points to fit all three parameters would result in overfitting. In general, the out-of-sample error of a model typically decays as 1/√n, where n is the number of data points. 
To reduce the error of the three fitted parameters due to stochastic noise in the nucleosome counts obtained in experiments, we used 6 levels of digestion (double of the minimum necessary number). This allowed us to capture the variability in the nucleosome digestion kinetics: some nucleosomes are released from most of the cells after 1 min of digestion, some after 2 min, other after 5 min, etc. Therefore, we recommend at least 5 levels of digestion in order to accurately fit the parameters (bottom p. 7). 
Since different cell types can have different nucleosome organizations, and different chromatin accessibility landscapes, it is impossible to accurately predict these properties using only the AT-content or other DNA-related properties. As described below, we observed very low correlations between two embryonic cell types, S2 and Kc167 cells, which emphasizes this difficulty. 
Nevertheless, using the AT-content in order to refine some of the three parameters analytically is an interesting idea that we tried to pursue. We tried multiple machine learning algorithms in order to predict the three parameters based on the sequence (and other features), but unfortunately we didn’t have sufficient success with this approach to include it in the manuscript. 
Regarding the tools to analyze similar experiments, we have provided the scripts used to process the data in this study on GitHub at: https://github.com/rchereji/qMNase-seq (p. 18, line 12).* 

2) It appears that this study is based on a single digestion series from a single drosophila cell line. In my opinion, it would be essential to repeat it at least one or two more times to be able to estimate whether variation observed in nucleosome occupancy are robust features of the different nucleosomes or simple variation between nucleosomes in this specific experiment. Comparing occupancy levels in two different cell lines/states would only be a nice addition, but not essential. 

*In the revised manuscript we have repeated the digestion series both in S2 cells and in a different cell line, Kc167. The positions of the called nucleosomes were very similar in the two experiments done using S2 cells. About 93% of all nucleosomes that were called in S2 cells have their positions (the median of each dyad cluster) shifted by less than 20bp in the two replicate experiments, and ~87% of all nucleosome were detected with positions shifted by less than 10bp. The nucleosome occupancies estimated from the two biological replicates agreed with each other, with a Pearson correlation coefficient of 0.787. However, comparing the nucleosome occupancies between S2 and Kc167 cells we obtained a Pearson correlation coefficient of only 0.362 (replicate 1) and 0.302 (replicate 2), indicating that the nucleosome occupancies are significantly different in these two cell lines, although the positions are similar.* 

3) It appears that the authors focused their analysis on "highly positioned" nucleosomes. While this was probably the most straightforward to do, I was wondering how this approach improves the measurement of nucleosome occupancy for less positioned nucleosomes. More importantly, how this selection might have influenced the conclusions drawn. In particular, I am not sure whether the proportion of positioned nucleosomes are the same in the different chromatin domains (Figure 9c) and if not is it safe to conclude that heterochromatin contains equally accessible nucleosomes as compared to other domains or this is based on a specific subset of positioned nucleosomes located in this domain. 

*We have extended our analyses to include all nucleosomes (not only the highly positioned nucleosomes). We modified our nucleosome calling algorithm to include nucleosomes characterized by clusters of reads with higher variance, and therefore, we are now calling ~682,000 (experiment 1) and ~678,000 (experiment 2) nucleosomes in S2 cells and ~687,000 nucleosomes in Kc167 cells. The total length of DNA that is covered by these nucleosomes is about 100 Mb, which represents about 73% of the whole genome size. This is in agreement with the fact that the nucleosome repeat length is about 200 bp, so the expected fraction of the genome that is covered by nucleosomes is ~147/200 = 73.5%. Therefore, we are confident that our nucleosome calling algorithm has detected most of the nucleosomes, including the poorly positioned ones. 
After extending the collection of called nucleosomes, and redoing all analyses for a second replicate experiment using S2 cells, and also for an additional cell line (Kc167 cells), the conclusions of the paper have remained unchanged, and are supported by multiple sets of experiments. For example, the bias in the AT-content of the mono-nucleosomal fragments obtained at different levels of digestion was also observed in the new experiments. 
The differences observed in the boxplots in Fig. 9 have increased slightly (see updated Fig. 9), because many of the nucleosomes that were less well-positioned (and not included in the first version) were located in the DHSs and on the highly transcribed genes, where nucleosome organization is disrupted by transcription and binding of other regulatory factors. Figure 9 shows the box plots corresponding to the first experiment done with S2 cells.* 

Minor points: 
- Figure 8 suggests that regions with vastly different occupancy values exist (10-80%), but it is not indicated what proposition of the genome/nucleosomes falls under these different categories and no further analysis presented to explore the reason for these massive differences. 

*We have included these proportions in the panels of the revised Fig. 8; each bin number is followed by the proportion of nucleosomes that are contained in each bin.* 

- Figure 6 suggest that digestion kinetics of neighboring nucleosomes are not entirely independent. If this is indeed the case, this would be worthwhile mentioning and perhaps discussing the effect of this on calculation of nucleosome occupancy. 

*Indeed, neighboring nucleosomes are not released from chromatin in an independent way, as they share a common linker. If a linker is AT-rich or particularly accessible to MNase, then both of the neighboring nucleosomes are expected to be released faster from chromatin, compared to other nucleosomes, that do not have any of the two linkers preferentially cut by MNase. 
We added the above observation to the main text (p. 11, lines 12-16), where we discuss Fig. 6.*

Reviewer #2: 
The authors provide a nice study aiming to offer a quantitative description of MNase-seq experiments. This is an uneasy but important task, and these results will be interesting to a number of scientists working in this area. The article is in general well-written and appropriate for Genome Biology. My suggestions are provided below in the order of the manuscript flow: 

1) Abstract: "Surprisingly, we observed no overall differences in nucleosome occupancies between Drosophila euchromatin and heterochromatin…" 
- I suggest to cite in the text in this respect a recent paper that also did not find differences in MNase accessibility between eu- and heterchromatin: https://www.ncbi.nlm.nih.gov/pubmed/24812327, as well as a paper from 1979 that compared the kinetics of MNase digestion in the intact chromatin vs in solution: https://www.ncbi.nlm.nih.gov/pubmed/489546. 

*We thank the Reviewer for bringing to our attention the two studies that we now cite in our paper.* 

2) Abstract: "…which implies that heterochromatic compaction does not reduce accessibility of linker DNA" 
- I would say that this statement should refer only to *MNase accessibility*, because some other proteins may still have different accessibility to linker DNA. 

*As suggested, we have changed that sentence to "…which implies that heterochromatic compaction does not reduce MNase accessibility of linker DNA".* 

3) Introductions: "If the number of nucleosomal fragments obtained 
from a given locus increases with the MNase concentration, then this locus is… called "inaccessible chromatin". If the number of nucleosomal fragments decreases with the MNase concentration… then this locus is … labeled as "accessible chromatin" 

- This interpretation is based on certain strong assumptions. Another way of looking at MNase-seq titration with different digestion levels that is less dependent on assumptions is to separate nucleosomes into "stable" and "labile" (MNase-sensitive) as was done previously: 
https://www.ncbi.nlm.nih.gov/pubmed/24812327. 

*In the paragraph mentioned by the Reviewer we only cited the study by Mieczkowski et al. and the definitions that those authors give for “accessible chromatin” and “inaccessible chromatin”, and we now make this clearer in the text. We also cited the reference indicated by the Reviewer as an alternative way of classifying the chromatin regions. As our aim is to determine occupancy, a fundamental property of nucleosomes in populations of living cells, we prefer not to use terms such as “stable” and “labile” that refer to behavior when the genome is fragmented, an assay that is profoundly non-physiological. Also, this binary distinction can be confused with what have been referred to as “fragile” nucleosomes (described below in response to Reviewer 3).* 

4) Results (pages 4 and 5): The computational method suggested here assumes that all MNase-seq digestion events across the genome are governed by the same biochemical equilibrium. It may be a proper assumption, but such assumption (and its limitations) needs to be written explicitly. For example, consider two locations X and Y. In location X there is a protein A sitting on the DNA and the DNA accessibility is determined by the kinetics of protein A dissociation. In location Y there is protein B sitting on the DNA that has a different dissociation kinetics. Similarly, some regions near the nucleosome dyad become accessible to MNase due to "nucleosome breathing" and this kinetics can be different in locations X and Y across the genome. 

*“Nucleosome breathing” affects mostly the first 1-2 helical twists from the edges of nucleosomes. We took this into account by counting as nucleosomes even the DNA fragments as short as 100 bp (which correspond to nucleosomes where ~2 helical twists have been destroyed by MNase on both sides of the nucleosome core). 
If other proteins protect DNA against MNase, their kinetics will be reflected in the 3 parameters that we fit for each location that gives a cluster of protected fragments. For example, if a transcription factor has a low residence time and a high dissociation rate, then we would obtain in our fit a low occupancy, a high accessibility (high release rate, k1) and a high decay rate (k2). A low occupancy can indicate two facts (equivalent if the system is ergodic): (i) a small fraction of the total population of cells have the corresponding region occupied by a nucleosome or another protein that protects DNA against MNase cleavage, at any given time; (ii) if a single cell is tracked during a long time interval, a small fraction of the total time, that region is bound and protected by a nucleosome or another protein. 
Moreover, other proteins that have very different dynamics compared to nucleosomes, do not affect the DNA protection in a significant way. After all, in order to see a peak in the MNase-seq occupancy profile, a protein has to be stably bound to DNA, and transient binding of fast transcription factors will not protect DNA during the course of 20-40-60 min of MNase digestion. Those transient binding events only give a strong signal in MNase-seq occupancy profiles during very short periods of time (e.g. 1 min digestion in our experiments). When we identified those sites that are bound by “MNase-sensitive” complexes, those accounted for a very small fraction of all called peaks, <1% of total peaks.* 

5) Equation 2: k1 and k2 are not defined in the text (although a definition appears in the legend of Figure 1). BTW, after noticing that k1 and k2 are defined as the functions of the location along the genome X in the legend of Figure 1, it seems that Equation 2 may need to be re-formulated by using explicitly the genomic coordinate X. It is also important to make the occupancy O(x) part of the equations in the main text. 

*We thank the Reviewer for pointing out the fact that k1 and k2 were not clearly defined in the main text and we clarified this in the revised manuscript. 
Although in the equations in the main text we have not written that all the parameters depend on the specific location “x” (in order to keep it simple in the main text), we started the paragraph by indicating that we analyze a specific locus “x”: 
“Suppose we start with a population of 𝐶 cells, and we focus on a particular genomic locus 𝑥” 
We clarified the dependence of O, k1, k2 on the locus x in the paragraph mentioned above and in the following paragraph, “Different genomic loci x are characterized by… nucleosome occupancy O(x), and the two rate constants k1(x) and k2(x)”. 
We refer the reader to the Supplementary Materials whenever more details are necessary, or further mathematical derivations are skipped in the main text.* 

6) Letter "N" is used to denote nucleosome occupancy in Equation 1. Is it the same as letter "O" in equation 2? Is the meaning of variable "N" used in Equation 2 different from the meaning of variable "N" in Equation 1? If so, it needs to be explained. 

*In Equation 1 we explained the procedure used by Mieczkowski et al. to define the MACC score. They use a linear fit for the “fragment frequency” as a function of the MNase units used in their digestion series. Their “fragment frequency” corresponds to the nucleosome counts, that we denoted by N in our manuscript, so the two N’s used in Equations 1 and 2 represent the same quantity: the number of mono-nucleosome reads obtained from a given locus. This read count measurement N is different from the letter “O”, which represents the calculated occupancy at a locus.* 

7) Looking through the notation explained in the Supplementary Materials it seems that N(t) and B(t) are defined there as concentrations, whereas N is mentioned as the number of nucleosomes or as the nucleosome occupancy in the main text. It is important to unify the notation with respect to all variables in all places in the main text and Supplementary Materials. 

*Conventionally, square brackets are used to denote concentrations. We have used the same convention throughout the manuscript. When we denote the number of nucleosomes (or mono-nucleosome reads obtained from the sequencer) we used the letter N, but when we write the reaction equations we use the symbol [N] to denote the concentration of released nucleosomes from each sample. These quantities are directly proportional to each other, and differ only by a simple multiplicative factor (the volume of each sample). We have checked again the main text and the supplement to make sure that the above convention was used consistently throughout the entire manuscript.* 

8) Results page 7: "we add equal amounts of spike-in DNA (S. cerevisiae mononucleosomal DNA obtained by a traditional MNase-seq method). 

- How were these mononucleosomes extracted from the MNase preparation? This information needs to be added to the methods section. 

*We have added this information to the Methods section.* 

9) Results page 7: "Then, we normalize the counts of fly mononucleosomes by the amount of spike-in DNA from each sample" 

- How exactly was the normalisation performed? What are the assumptions here? 

*We added yeast spike-in DNA proportional to the number of cells from each tube (proportional to the amount of fly DNA). Therefore, by normalizing the number of sequencing reads such that all samples have the same number of spike-in fragments, we effectively counted the number of nucleosomes that were released from the same number of cells (p. 7, line 9 from bottom). 
For example, let’s assume the following scenario. Suppose samples A and B contained in the beginning the same number of cells, and the same amounts of spike-ins. If sample A is only mildly digested and results in 4 times less mono-nucleosomal DNA compared to sample B, then during the library preparation perhaps we need to do 2 extra cycles of PCR in order to obtain similar sequencing libraries. These extra PCR cycles will produce 4 times more spike-in DNA fragments being sequenced from sample A compared to sample B, and in the end, by normalizing to have the same number of spike-ins in each sample, we will find that sample A had 4 times fewer mono-nucleosomes released from chromatin compared to sample B. 
This normalization procedure is illustrated in Figure S4: black and red bars indicate the number of DNA fragments obtained from fly and spike-ins. After normalization, all samples contain the same amount of sequenced spike-ins, indicating that the normalized numbers of fly mono-nucleosomal fragments originated from equal numbers of cells.* 

10) Figure 8: Please add the best fit parameters for k1 and k2 to each panel. 

*In Figure 8, we binned the loci according to the fitted nucleosome occupancy. Therefore, each bin (panel) contains profiles fitted using a wide range of k1 and k2 values (each locus has its own set of 3 fitted parameters: O, k1 and k2), and there is no unique/best k1/k2 for the entire bin.* 

11) Figure 9: It would be informative to show also the values of k2 and the average nucleosome occupancy for each case. 

*We have updated Fig. 9 and now it includes additional panels showing the distributions of k2 and nucleosome occupancy in each of the groups.*

Reviewer #3: 
The authors submitted a very good protocol to quantitatively analyse nucleosome occupancy. As chromatin of different genomic regions exhibits different MNase sensitivity and accessibility, nucleosomes are released at different rates. The authors provide a model and equation to quantify these effects, taking into account that nucleosome cores released early are prone to their disruption by hydrolysis of the nucleosomal DNA. They used Drosophila S2 cells to prepare a time course of MNase hydrolisation, in order to test the proposed model. 
MNase is a widely used tool that exhibits a high preference for the AT-content of DNA, thereby preferentially hydrolysing AT-rich linker DNAs and releasing nucleosomes reconstituted on AT-rich genomic regions first. In turn these AT-rich nucleosomes could be also more susceptible to hydrolysis by MNase and being the first ones to be destroyed. 
The authors can convincingly show the early release of nucleosomes at AT-rich DNA sequences and observe the subsequent elimination of these nucleosomal particles out of the pool. Increasing hydrolysis time results in nucleosomal and sub-nucleosome sized protected DNA with decreasing AT-content. These effects are not new, but the quantitative description of these effects has its merit. 
The addition of the spike-in DNA to normalise the samples is essential for quantitative assessment and the color coding of the called nucleosomes according to their time point of maximal intensity is very appealing. The protocol should serve as a best practice example to plan and analyse MNase-seq data. 
However, as MNase-seq requires deep sequencing of the nucleosomal DNA, the preparation of six libraries and sequencing reactions would make this kind of experiments very expensive for large genomes. As the authors want to establish a general MNase-seq pipeline, they should address the question of how many time points are essential to reveal qualitative different nucleosome species, and/or their association with epigenetic marks/DNA sequence features/regulatory elements/etc.. Would the first and last time point of MNase hydrolysis be sufficient to obtain the same results, or are three time points sufficient? 

*For a qualitative description, a mild and an extensive digestion is enough to separate the nucleosomes that are the most accessible to MNase (and MNase-sensitive) from the nucleosomes that are less accessible, and which remain in chromatin until the latest stages of digestion. This kind of analyses, comparing a mild digestion with an extensive digestion, have been done in the past and have contributed to the controversy about fragile nucleosomes (e.g. Chereji et al. (2017) Mol Cell 65:565-577; Kubik et al. (2017) Mol Cell 65:578-580; Brahma & Henikoff (2019) Mol Cell 73:238-249). 
In our study, we did a quantitative analysis and modeled the nucleosome release and decay by MNase and tried to clarify that nucleosomes should not be classified in a binary way as fragile and non-fragile (or stable). Different nucleosomes are made of different DNA sequences, and all these sequences are digested by MNase at different rates. So instead of only two cases (fragile vs. stable), nucleosomes are characterized by a broad range of MNase-sensitivities, depending on the individual DNA sequence and the corresponding linkers that flank each nucleosome. Moreover, the chromatin accessibility modulated by histone marks, transcription, etc. adds an additional layer of complexity, and DNA sequence alone is not the only determinant of the rates of nucleosome release. 
For estimating the three parameters that characterize each nucleosome (occupancy and k1, k2 rate constants) a minimum of 3 data points is necessary (in the ideal case, if noise were absent). Given that the numbers of sequencing reads are affected by stochastic noise, in practice, 3 data points are not enough to estimate the 3 parameters in an accurate way for all nucleosomes (see the first answer to Reviewer 1). We decided to double the number of time points in order to improve the accuracy of the estimations (p. 7 bottom).* 

I think that with the rigorous analysis of the MNase-seq data the authors provide an excellent protocol. However, besides this, the manuscript provides only incremental new insights to chromatin organisation. The finding that the MNase accessibility is similar for euchromatin and heterochromatin was previously published for human cells by Schwartz and Längst in Nucleic Acids Research (2019). Surprisingly the authors do not cite or discuss this manuscript. The methods are similar, however, Schwartz and colleagues use only two time points of MNase hydrolysis that are sufficient to draw the same conclusion. For this I think it is important to address the minimal number of digestion points, as described above. 

*We thank Reviewers 1 and 3 for pointing out the recent study by Schwartz and Längst, which we cite in the revised manuscript. 
There is a tradeoff between the number of data points (i.e. digestions) that we measure, and the accuracy of the predicted parameters. It is similar with the case of estimating the slope of a linear fit in a simple scatter plot. We can use a minimum of 2 points to fit a linear function, but that represents an overfit. The more data points we use to fit a model, the better we estimate the slope, and reduce the out-of-sample error, which typically decays as square root of the number of data points. 
To illustrate this fact, we added in the Supplement a new section “Estimation of the errors” in which we simulated noisy data and fitted back the three parameters using 5, 10, and 15 data points. We show that the relative error of the parameters is reduced by using more data points to fit the parameters.* 

Having this quantitative evaluation of nucleosome occupancy. Is it possible to normalise a genome for the AT-content and to provide a measure for an optimal degree of MNase digestion to the experimentalists? In order to have a golden standard for future MNase-seq experiments, this recommendation would be highly valuable for chromatin structure analysis. 

*Unfortunately, a single MNase digestion is not enough to obtain the true nucleosome occupancy, or at least a proportional measure of the occupancy. As we show in Equation 2, the number of released nucleosomes is not directly proportional to the nucleosome occupancy (it cannot be written as a constant x Occupancy), but it also depends on the level of digestion (MNase concentration [E] and digestion time t), and on the rates of nucleosome release (k1) and nucleosome decay (k2) - see Eq. 2. 
Therefore, no matter how one finetunes the condition of digestion, the factor 
k_1/(k_1-k_2 )(e^(-k_2 [E]t)-e^(-k_1 [E]t)) 
from Equation 2 is still variable, given that k1 and k2 vary from locus to locus in a complex way. This factor depends on the specific DNA sequence, on linker sequences, and chromatin accessibility, and so it varies at different loci, in different cell types, and different conditions (e.g. if a gene is activated, the DNA accessibility might change). Consequently, the number of mono-nucleosomal fragments obtained at any level of digestion is not a good measure for the nucleosome occupancy, as these quantities are not directly proportional, i.e. N is not just a constant time occupancy.*

**Second round of review**

**Reviewer 2**

The authors have addressed all my points.

**Reviewer 3**

The authors addressed all the questions raised by the reviewers. They also included additional datasets to show the reproducibility and robustness of their protocol. 
The only change I request, is a rephrasing of the abstract. The authors write "Surprisingly, we observed no overall differences in nucleosome occupancies between Drosophila euchromatin and heterochromatin..", however this finding is not surprising, as it was previously published (a manuscript they cite in the discussion). It would be correct to say that like in human cells no overall differences in nucleosome occupancies between euchromatin and heterochromatin can be observed in Drosophila. They should change the abstract accordingly.
Besides that the manuscript does not require additional changes.
